# Next generation sequencing for gut microbiome characterization in rainbow trout (*Oncorhynchus mykiss*) fed animal by-product meals as an alternative to fishmeal protein sources

**DOI:** 10.1371/journal.pone.0193652

**Published:** 2018-03-06

**Authors:** Simona Rimoldi, Genciana Terova, Chiara Ascione, Riccardo Giannico, Fabio Brambilla

**Affiliations:** 1 Department of Biotechnology and Life Sciences, University of Insubria, Varese, Italy; 2 Inter-University Centre for Research in Protein Biotechnologies "The Protein Factory"- Polytechnic University of Milan and University of Insubria, Varese, Italy; 3 Fondazione Parco Tecnologico Padano, Lodi, Italy; 4 VRM S.r.l. Naturalleva, Cologna Veneta, Verona, Italy; Universidade de Vigo, SPAIN

## Abstract

Animal by-product meals from the rendering industry could provide a sustainable and commercially viable alternative to fishmeal (FM) in aquaculture, as they are rich in most essential amino acids and contain important amounts of water-soluble proteins that improve feed digestibility and palatability. Among them, poultry by-product meal (PBM) have given encouraging results in rainbow trout (*Oncorhynchus mykiss*). However, the introduction of new ingredients in the diet needs to be carefully evaluated since diet is one of the main factors affecting the gut microbiota, which is a complex community that contributes to host metabolism, nutrition, growth, and disease resistance. Accordingly, we investigated the effects of partial replacement of dietary FM with a mix of animal by-product meals and plant proteins on intestinal microbiota composition of rainbow trout in relation to growth and feeding efficiency parameters. We used 1540 trout with an initial mean body weight of 94.6 ± 14.2 g. Fish were fed for 12 weeks with 7 different feed formulations. The growth data showed that trout fed on diets rich in animal by-product meals grew as well as fish fed on control diet, which was rich in FM (37.3%) and PBM-free. High-throughput 16S rRNA gene amplicon sequencing (MiSeq platform, Illumina) was utilised to study the gut microbial community profile. After discarding *Cyanobacteria* (class *Chloroplast*) and mitochondria reads a total of 2,701,274 of reads taxonomically classified, corresponding to a mean of 96,474 ± 68,056 reads per sample, were obtained. Five thousand three hundred ninety-nine operational taxonomic units (OTUs) were identified, which predominantly mapped to the phyla of *Firmicutes*, *Proteobacteria*, *Bacteroidetes* and *Actinobacteria*. The ratio between vegetable and animal proteins proved to play a central role in determining microbiome profiles and *Firmicutes* and *Proteobacteria* phyla were particularly discriminatory for diet type in trout. Plant ingredients favoured a higher *Firmicutes*:*Proteobacteria* ratio than animal proteins. Acceptable abundance of *Firmicutes* was guaranteed by including at least 25% of vegetable proteins in the diet regardless of animal protein source and percentage. In summary animal by-product meals, as replacements to FM, gave good results in terms of growth performances and did not induce significant changes in gut microbial richness, thus proving to be a suitable protein source for use in rainbow trout aqua feed.

## Introduction

In aquaculture, feed accounts for over 50 percent of the production cost. This high cost is in large part due to the use of expensive ingredients such as fishmeal (FM) and fish oil (FO). Shepherd and Jackson (2013) [1] gave a detailed picture on the global volumes of production and consumption of FM and FO for the period 2001–2011, based on IFFO and Oil World data. The nutritive value of fish feed depends on the quality of the proteins used. For this, FM is a preferred ingredient, in particular for carnivorous species, such as salmonids. However, limited availability and high price of FM have driven the aquafeed industry to look for alternative protein sources in order to satisfy the demand of the continuously growing aquaculture sector [2,3].

Rational use of limited marine protein sources and development of nutritionally adequate feed formulations based on more readily available and economical alternative protein ingredients, are thus required [4–6]. In the last few years, significant advances have been made in this direction; currently, some commercial fish feeds can contain even less than 10% of FM. The most commonly used alternatives to expensive FM are of plant origin, such as oilseed meals (soybean, canola, and sunflower), grains (wheat and corn), and legumes (lupine, bean, and peas) [7]. Nevertheless, several nutritional issues are associated with the utilization of plant ingredients, due to their unbalanced amino acid profile and to the presence of anti-nutritional factors (ANFs) [8–11]. In plant feedstuffs, ANFs include indigestible components such as fibers, phosphorous-rich phytic acid, saponins, and protease inhibitors [10] that may reduce fish feed intake, growth, nutrient digestibility and utilization, and alter disease resistance, thus leading to poor fish growth [8, 12–14]. Therefore, it is crucial to find appropriate protein sources alternative to FM for aquafeed production. In this regard, some recent studies have shown that animal by-product meals, arising from the rendering industry, could be suitable for use as dietary FM replacers [15–18]. Unlike plant proteins, animal proteins are rich in most essential amino acids and contain important amounts of water-soluble proteins, which, besides being highly digestible, also improve feed palatability [16,17,19,20]. In recent years, after previous bans following the outbreak of the transmissible spongiform encephalopathy [21], the European Union has re-authorized the use of non-ruminant animal by-product meal (meat meal, blood meal, poultry by-product meals, and hydrolyzed feather meal) in aquafeeds. Since June 2013, it is thus possible to partially replace FM with different blends of non-ruminant animal proteins. Of these, one of the most promising and attractive options for fish feed formulations is poultry by-product meal (PBM), which consists of rendered clean parts of the poultry carcass such as neck, head, feet, undeveloped eggs, gizzard, and intestine [22]. PBM is generally a palatable, high-quality protein source due to its proper balance of essential amino acids, fatty acids, vitamins, and minerals [17,23].

The use of PBM in several fish species such as grass carp (*Ctenopharyngodon idellus*), turbot (*Scophthalmus maximus*), Nile tilapia (*Oreochromis niloticus*), and rainbow trout (*Onchorynchus mykiss*) has had positive effects on feed palatability, fish survival rate, growth performances, and protein retention [16,17,20,24–27].

However, the introduction of new ingredients in the diet needs to be carefully evaluated since diet is one of the main factors putting selective pressure on the gastrointestinal microbial composition in vertebrates, including fish [28]. Several studies in humans and mammals have undoubtedly correlated gut microbial communities with host physiology, nutrition, and growth [29–31]. Like in mammals, the intestinal microbiota of fish has important functions in host metabolism, mucosal development and maturation, nutrition, immunity, and disease resistance [32–35]. Fish gut microbiota is responsible for the synthesis of vitamins, digestive enzymes, and metabolites such as short-chain (volatile) fatty acids that represent the main energy source for intestinal epithelial cells [28,35–38]. Furthermore, fish intestine harbors a wide range of bacteria, mainly lactic acid bacteria, that can inhibit bacterial pathogens by secreting antimicrobial compounds such as lactic and acetic acids [28,39]. On the other hand, an imbalanced microbiota could negatively affect fish nutrition and growth and lead to an alteration of gut immune functions contributing thus to the development of diseases. Therefore, a better understanding of gut/microbe interactions and gut microbial diversity in fish could be highly relevant for aquaculture practice.

Fish microbiota has traditionally been studied by culture methods and subsequent identification based on biochemical and phenotypic characteristics of bacteria. However, culture-dependent techniques give a limited picture of intestinal microbiota because only a low fraction, down to about 1% of the bacteria from fish intestine can be cultivated. Therefore, in the last few years, several culture-independent molecular techniques have been developed and applied to studies of fish gut microbiota [35]. The most powerful approach to study the composition of complex intestinal microbial communities is represented by Next-generation Sequencing (NGS) technology [37,40]. Metagenomic profiling by high-throughput sequencing of 16S rRNA or cpn60 gene, was applied in some recent studies to investigate the impact of dietary plant ingredients on fish gut microbiota composition [38,41–44]. In rainbow trout, for example, a diet containing proteins from terrestrial plants such as pea and soy generally led to a higher *Firmicutes*:*Proteobacteria* ratio than a FM-based diet [44]. Conversely, replacing FM with a mixture of plant meals in the diet of sea bream (*Sparus aurata*) had a negative effect on the relative abundance of *Firmicutes* phylum throughout the gut, in particular, on lactic acid bacteria belonging to genera *Streptococcus* and *Lactobacillus* [42]. Whereas several studies have thoroughly investigated the effects of plant-based diets on fish gut microbiota composition, only a limited number of researches have been focused on the effects of FM replacement with animal by-product meals [45].

Accordingly, the present study aimed to investigate, for the first time, the effect of replacement of FM with seven different blends of terrestrial animal and plant proteins on intestinal microbiota of rainbow trout, trying to correlate any changes in microbial communities’ profile to the performance outcomes of fish. The Illumina MiSeq platform for high-throughput sequencing of 16S rRNA gene was utilized to analyze and characterize the whole gut microbiome of trout fed with five different experimental formulations and two commercial feeds. Our assumption was that animal by-product meals could not negatively affect intestinal microbial profile of rainbow trout being thus a valid alternative to FM in feed formulation.

## Materials and methods

### Ethics statement

This study was carried out in strict accordance with the recommendations in the Guide for the Care and Use of Laboratory Animals of the Edmund Mach Foundation (F.E.M) (San Michele all'Adige, Trento, Italy), and in accordance with EU Directive 2010/63/EU for animal experiments. The Committee on the Ethics of Animal Experiments of the same Foundation approved all of the protocols performed [approval n. 120/2008-A of 03/09/2008 (Art.12 of D.Lgs.116/92). Fish handling was performed under tricaine methanesulfonate (MS222) anesthesia, and all efforts were made to minimize discomfort, stress, and pain to the fish.

### Fish, rearing conditions, and diets

All procedures involving rainbow trout (*O*. *mykiss*) were conducted at the indoor experimental facility of Edmund Mach Foundation (F.E.M) (San Michele all'Adige, Trento, Italy).

We used 1540 trout (13 months old, all female) with an initial mean body weight of 94.6 ± 14.2 g and a total length of 21.4 ± 1.2 cm. Fish were randomly distributed into 14 fiberglass tanks of 3600 litres (110 fish/tank, at a rearing density of 2,89 kg/m^3^) connected to a flow-through fish rearing system. Experimental tanks were supplied with degassed ground water with an approximately constant temperature of 12.5 ± 0.3°C and dissolved oxygen concentration at 9.1 ±0.6 mg/l (DO saturation over 85%). Fish were acclimatized for six days under natural photoperiod and fed to visual satiety with a standard commercial diet (VRM S.r.l, Naturalleva, Italy). After the acclimation period, fish were fed twice daily for 12 weeks with seven different extruded diets (4.5 mm diameter pellets) in duplicate (2 tanks/diet). Five diets (A-E) were formulated specifically for this study by Naturalleva (VRM S.r.l Italy), whereas diets F and G were commercial feeds manufactured by competitors. We have reported the proximate composition of all the diets in Table 1 and the formulation of the experimental diets (A-E) in Table 2. In the first four experimental diets (A-D) (Tables 1 and 2), FM was partially replaced by different mixtures of plant and animal by-product proteins, i.e. poultry by-product meal (PBM) and porcine blood meal. The latter ones derived from animals, which passed as fit for human consumption under veterinary supervision, before their slaughter. In particular, diets A and B had a discrete content of FM and high levels of animal by-product and plant proteins. Diets C and D had a higher percentage of animal by-product meals and a lower percentage of FM than the two previous diets, but the highest content of plant proteins. Diet E (control) contained only FM, porcine blood meal, and vegetable meal as protein sources (no PBM) (Tables 1 and 2). Diets F and G (commercial feeds manufactured by competitors) were instead characterized by the highest percentage of animal by-product meals, most of them deriving from PBM (Table 1). The information provided in the labels of these two feeds are the followings. Diet F: PBM, FM, wheat, fish oil, vegetable oils (soybean, rapeseed), porcine blood meal, vegetable meal (dehulled soybean and sunflower), wheat meal, volatile blood meal, vitamin, mineral, and antioxidant premixes. Diet G: PBM, vegetable meal (wheat, dehulled soybean), hydrolysed feather meal, FM, rapeseed oil, fish oil, porcine blood meal, sunflower seed meal, soybean oil, guar germ meal, vital wheat gluten. vitamin, mineral, and antioxidant premixes.

**Table 1:** Proximate composition (g ∙ kg^-1^ diet) and amount (%) of different protein sources used for the formulation of the experimental diets.

	DIET						
	A	B	C	D	E	F	G
Moisture	70.0	70.0	70.0	70.0	70.0	70.0	70.0
Crude protein	410.0	420.0	410.0	420.0	430.0	420.0	430.0
Crude lipids	260.0	240.0	240.0	180.0	260.0	280.0	220.0
Crude fiber	20.0	21.0	28.0	28.0	13.0	18.0	30.0
NFE	175.0	184.0	187.0	237.0	162.0	128.0	180.0
Ash	65.0	65.0	65.0	65.0	65.0	84.0	70.0
Phosphorus	5.5	5.4	5.1	5.4	4.7	4.6	3.8
GE (MJ kg^-1^)	23.0	22.5	22.5	21.1	23.2	23.3	22.0
**Relative amount of different protein sources (%)**:							
FP/TP	20.6	20.9	10.6	11.2	37.3	20.0	11.0
TAP/TP	68.0	68.0	64.0	56.0	62.0	75.0	80.0
AP (TAP-FP)	47.4	47.4	53.4	44.8	24.7	55.0	69.0
VP/TP	32.0	32.0	36.0	44.0	38.0	25.0	20.0

**NFE**: Nitrogen-free extract; **GE**: gross energy (calculated using combustion values for protein, lipid and carbohydrate of 23.6; 39.5; and 17.2 MJ/kg, respectively); **FP**: fish proteins; **TP**: total proteins; **TAP**: total animal proteins; **AP**: animal proteins from alternative sources; **VP**: vegetable proteins.

**Table 2:** Formulations of the experimental diets (in percentage).

	A	B	C	D	E
Fish meal	13.49	13.84	6.92	7.21	26.06
Dried swine hemoglobin	0.00	0.00	0.00	0.00	4.25
Dried swine plasma	12.01	12.32	12.32	11.12	8.16
Poultry by-products meal	12.71	13.04	15.54	12.60	0.00
Fish oil	16.39	14.90	3.61	2.42	16.02
Rapeseed meal	6.86	7.04	12.32	8.95	0.00
Soybean meal	6.65	6.82	15.72	10.95	7.30
Guar germ meal	2.57	2.64	0.00	10.68	4.79
Wheat flour	6.73	6.90	5.07	10.71	7.38
Corn gluten	0.00	0.00	0.00	0.00	3.36
Vital wheat gluten	3.35	3.44	0.00	0.00	0.00
Peas	11.93	12.24	10.98	12.95	9.60
Soy protein concentrate	0.00	0.00	0.00	0.00	6.59
Soybean oil	5.51	5.04	15.84	10.74	5.47
DL- methionine	0.55	0.56	0.40	0.44	0.31
Lisin	0.33	0.30	0.29	0.20	0.10
Taurin	0.30	0.32	0.40	0.43	0.00
Antioxidants premix^a^	0.06	0.04	0.03	0.04	0.05
Vitamin and mineral premix^b^	0.50	0.50	0.50	0.50	0.50
Stay C 35%	0.06	0.06	0.06	0.06	0.06
	100.0	100.0	100.0	100.0	100.0

^a^ Propyl Gallate: 9.9%; B.H.A.: 5.0%, Ethoxyquin: 9.9%; Citric acid: 11.0%; Carrier (= SiO2) ad 100%.

^b^ Vitamin and mineral premix (quantities in 1 kg of mix): Vitamin A, 4,000,000 IU; Vitamin D3, 800,000 IU; Vitamin C, 25,000 mg; Vitamin E, 15,000 mg; Inositol, 15,000 mg; Niacin, 12,000 mg; Choline chloride, 6,000 mg; Calcium Pantothenate, 3,000 mg; Vitamin B1, 2,000mg; Vitamin B3, 2,000mg; Vitamin B6, 1,800 mg; Biotin, 100 mg; Manganese, 9,000 mg; Zinc, 8,000 mg; Iron, 7,000 mg; Copper, 1,400 mg; Cobalt, 160 mg; Iodine 120 mg; Anticaking & Antioxidant + carrier, making up to 1000 g.

Fish feeding rates were restricted to 1.5% of biomass during the feeding trial. To calculate feed ratio, individual weight of 30 randomly chosen fish per tank (60 fish/diet) was assessed at 14, 42, and 70 days from the beginning of the trial, whereas all fish in the tank (220 fish/diet) were measured for their weight and body length at the beginning and the end of the experiment. Fish growth performance data were used as basis for the calculation of feed conversion ratio (FCR = dry feed intake/wet weight gain), condition factor [K = 100 (wet weight (g)/total length (cm)^3^], and specific growth rate [SGR (%/day) = 100 x [ln (final body weight)–ln (initial body weight)]/days], for each dietary fish group.

### Amino acid profile of diets

Total amino acid composition of each diet was determined by a Jasco HPLC system (Jasco-Europe S.r.l) consisting of a quaternary pump (Model PU-2089, Jasco) connected to a degasser, a programmable fluorescence detector (Model FP-4025, Jasco) (excitation 250 nm, emission 395 nm) and a temperature control module. The amount of sample used was 100 mg, which contained approximately 5 mg of crude protein that were hydrolyzed with 6 M HCl at 110–120°C for 22–24 h.

L-α-amino-*n*-butyric acid (Sigma Aldrich, Italy) was added as an internal standard before hydrolysis. Methionine (Met) and tryptophan (Trp) were determined separately. For Met quantification, performic acid oxidation followed by acid hydrolysis was used, whereas for Trp quantification, the procedure consisted of hydrolysis in 4.2 M NaOH at 100°C for 4 h, followed by neutralization of hydrolysate, and dilution in ultrapure water. After borate buffer addition and filtration, amino acids were derivatized with AccQ-Fluor Reagent Kit (6-aminoquinolyl-N-hydroxysuccinimidyl carbamate, Waters S.p.A., Italy) at 55°C for 10 min and injected in HPLC. Amino acids separation was performed by using a C-18 reverse-phase column Waters Acc. Tag (150 mm × 3.9 mm) (Waters, Italy) and a Phenomenex pre-column filter according to Liu et al. [46]. Briefly, the column was heated at 37°C for total amino acids and at 31°C for sulphur containing amino acids (Met), and Trp. The flow rate was fixed at 0.8 ml/min, mobile phase A consisted of acetate-phosphate aqueous buffer, mobile phase B of acetonitrile 100% and phase C was ultrapure water. The amino acid composition of each experimental diet is reported in Table 3.

**Table 3:** Amino acid composition (g · kg^-1^ diet) of the experimental diets.

	DIET						
	A	B	C	D	E	F	G
Alanine	27.4	27.1	25.1	24.7	27.2	26.4	27.7
Arginine	25.1	26.1	23.7	25.3	27.3	26.4	25.4
Aspartate	36.4	37.2	34.0	34.9	39.7	38.4	37.1
Glutamic Acid	50.5	52.9	50.9	54.3	57.9	56.1	54.7
Glycine	34.6	34.2	31.0	31.9	28.1	27.2	34.1
Histidine	13.0	12.7	12.3	11.5	13.5	13.1	13.6
Isoleucine	13.2	14.3	13.5	14.8	13.0	12.6	14.1
Leucine	33.7	34.4	32.7	32.0	36.3	35.1	35.5
Lysine	27.7	27.2	22.9	22.5	28.0	27.1	27.8
Methionine	9.9	10.2	8.4	9.6	10.1	9.8	10.4
Phenylalanine	19.9	20.4	19.2	19.0	21.7	21.1	20.9
Proline	28.0	29.0	27.1	28.2	21.6	20.9	29.0
Serine	24.7	26.2	24.3	25.1	20.0	19.3	25.7
Threonine	17.1	17.6	16.6	16.7	18.0	17.4	17.8
Tyrosine	10.9	11.4	10.6	11.0	14.0	13.5	11.5
Tryptophan	4.3	4.3	4.0	3.9	4.8	4.6	4.4
Valine	23.6	24.2	23.1	22.5	22.8	22.1	24.8

### Fatty acid profile of diets

The fatty acid composition of each diet is listed in Table 4. Total lipids were extracted according to Folch et al. [47] by using dichloromethane instead of chloroform. Following lipid extraction, fatty acid methyl esters (FAME) were prepared by acid-catalyzed transmethylation of total lipids using boron trifluoride (BF_3_) in methanol according to Santha and Ackman [48] and then analyzed by gas chromatography. The individual fatty acids were identified by comparing their retention times to that of standard FAME mixture (Supelco 37 Component FAME mix, Sigma Aldrich, Italy) and their relative proportions determined

**Table 4:** Fatty acid composition (% total fatty acids) of the experimental diets.

	DIET						
	A	B	C	D	E	F	G
12:0	0.02	0.02	0.01	0.02	0.02	0.02	0.02
14:0	1.86	1.79	0.72	0.76	1.99	1.97	1.76
15:0	0.15	0.15	0.06	0.07	0.16	0.16	0.14
16:0	10.37	9.97	10.62	10.69	9.90	9.89	10.20
17:0	0.18	0.17	0.13	0.14	0.18	0.18	0.17
18:0	3.19	3.07	3.95	3.94	2.95	2.95	3.16
20:0	0.48	0.46	0.36	0.35	0.47	0.48	0.46
22:0	0.34	0.33	0.37	0.38	0.28	0.28	0.35
24:0	0.07	0.07	0.07	0.06	0.07	0.07	0.07
25:0	0.02	0.02	0.04	0.04	0.02	0.02	0.02
14:1	0.02	0.02	0.01	0.02	0.01	0.01	0.02
16:1	2.27	2.19	1.00	1.13	2.29	2.26	2.21
17:1	0.14	0.13	0.08	0.09	0.14	0.14	0.13
18:1	0.01	0.01	0.01	0.01	0.01	0.01	0.01
18:1n-7	0.64	0.62	0.70	0.70	0.63	0.64	0.67
18:1n-9	28.82	27.61	24.28	24.01	27.26	27.53	28.14
20:1n-9	3.66	3.51	0.95	0.97	3.61	3.65	3.44
22:1n-9	0.53	0.50	0.11	0.11	0.54	0.55	0.49
22:1n-11	1.49	1.42	0.44	0.45	1.49	1.50	1.36
24:1n-9	0.09	0.09	0.03	0.03	0.09	0.09	0.09
18:2n-6	23.43	22.44	40.79	38.86	21.64	21.99	22.99
18:3n-6	0.20	0.19	0.08	0.08	0.20	0.20	0.19
20:2n-6	0.67	0.64	0.16	0.17	0.65	0.66	0.63
20:3n-6	0.26	0.25	0.06	0.06	0.25	0.26	0.25
20:4n-6	0.25	0.25	0.14	0.17	0.24	0.23	0.26
22:2n-6	2.3	2.2	0.6	0.6	2.3	2.3	2.1
18:3n-3	9.42	8.99	6.12	5.83	9.12	9.28	9.00
18:4n-3	0.64	0.61	0.22	0.24	0.68	0.68	0.60
20:3n-3	0.05	0.05	0.02	0.01	0.05	0.05	0.05
20:4n-3	0.32	0.30	0.11	0.11	0.33	0.33	0.29
20:5n-3	2.20	2.12	0.80	0.86	2.51	2.47	2.07
22:5n-3	0.68	0.65	0.23	0.24	0.71	0.71	0.63
22:6n-3	3.02	2.91	1.12	1.24	3.33	3.27	2.82
Σn-3PUFA	16.59	16.26	9.11	9.10	17.42	17.42	16.19
Σn-6PUFA	25.00	24.77	42.30	40.52	24.09	24.33	25.57
n-3/n-6	0.66	0.66	0.22	0.22	0.72	0.72	0.63
DHA/EPA	1.37	1.37	1.40	1.44	1.32	1.32	1.36

### Sampling

At the end of the feeding trial, two fish from each tank (4 fish/diet) were caught and euthanized with an overdose (320 mg/L at 22 °C) of anesthetic (tricaine-methasulfonate MS-222, Sigma-Aldrich, Italy). External surface of each fish was wiped with 70% ethanol to avoid the contamination of gut content by the external body surface microflora during dissection. With the aid of sterile scissors and forceps, the entire intestine (excluding pyloric caeca) was exposed from the ventral side, and then aseptically removed from each individual fish. The faecal content was obtained by squeezing out and scrapping the intestinal mucosa with a sterile spatula, in order to collect the luminal and the mucosa-associated microbiota. The faecal samples were collected in sterile tubes, immediately frozen in dry ice and then stored at—80°C until analysis.

### DNA extraction

We extracted total bacterial genomic DNA from all the collected faecal samples and used it as template in the 16S rRNA gene PCR amplification. Briefly, 1g of faeces from each fish was shaken with 5 ml of ASL buffer provided in the QIAamp DNA Stool Mini Kit (Qiagen, Italy). Then, 2 ml of homogenate were transferred into a microcentrifuge tube with two 5-mm stainless steel beads and then shaken on a TissueLyser II (Qiagen, Italy) for 5 min at 20 Hz. A sample of 2 ml of ASL buffer was processed in parallel as a negative control to check that no external DNA contamination was introduced during the extraction procedure. Bacterial DNA was then extracted according to the manufacturer's instructions. DNA concentration was measured by both, NanoDrop^™^ 2000 Spectrophotometer (Thermo Scientific, Italy) and Tecan Microplate Reader using Quant-iT™ PicoGreen^®^ dsDNA Assay Kit (Thermo Scientific, Italy). The extracted DNA samples were then diluted to a concentration of 5 ng/μl.

### Intestinal microbiome analysis

#### 16S rRNA gene amplicon sequencing library preparation

The Illumina protocol “16S Metagenomic Sequencing Library Preparation” (#15044223 rev.B) was applied to prepare 16S ribosomal RNA gene amplicons for Illumina MiSeq system. The variable V3 and V4 regions of the 16S rRNA gene were amplified from bacterial DNA obtained from fish faecal samples. The PCR reactions were performed using the 16S amplicon PCR forward primer (5’ CCTACGGGNGGCWGCAG 3’) and reverse primer (5’ GACTACHVGGTATCTAATCC 3’), which were selected by Klindworth et al. [49] as the most promising bacterial primer pair. Illumina adapter overhang nucleotide sequences were added at the 5’ end of both primers. PCRs were carried out in 25-μl reactions containing 2.5 μl of microbial DNA (12.5 ng), 5 μl of each primer (1μM), and 12.5 μl of 2X KAPA Hifi HotStart Ready Mix (Kapa Biosystems Ltd, UK). A no template control, in which nuclease free water was added instead of bacterial DNA, and a negative control, with the extraction from the sample containing ASL buffer only, were included in this PCR. Reaction times and cycling conditions were 95°C for 3 min, 25 cycles of 95°C for 30 s, 55°C for 30 s, 72°C for 30 s, and 72°C for 5 min. The resulting PCR products were run on an Agilent 2200 TapeStation (Agilent Technologies, Italy) to verify the size. The expected size of amplicons was about 550 bp. The PCR products were then purified from primers and primer dimers using Agencourt AMPure XP Kit (Beckman Coulter Genomics, Italy). Dual indices and Illumina sequencing adapters (P5 and P7) were then attached to the amplicons using Nextera XT Index Kit (Illumina, San Diego, CA) to produce the final libraries. The index PCRs were carried out in 50-μl reactions containing 5 μl of DNA, 5 μl of Nextera XT Index Primer 1, 5 μl of Nextera XT Index Primer 2, 25 μl of 2x KAPA Hifi HotStart Ready Mix (Kapa Biosystems Ltd, UK), and 10 μl of nuclease-free water. The PCR reaction conditions were the followings: 95°C for 3 min, 8 cycles of 95°C for 30 s, 55°C for 30 s, 72°C for 30 s, and 72°C for 5 min. Before quantification, the libraries were cleaned up using AMPure XP beads (Beckman Coulter Genomics, Italy) and the size of amplicons was verified on Agilent 2200 TapeStation (Agilent Technologies, Italy). The expected size of the final library was ~630 bp. Final libraries were quantified by absolute, quantitative PCR (qPCR) using KAPA Library Quantification Kits for Illumina^®^ platforms (Kapa Biosystems Ltd, UK). In particular, library quantification was performed by amplifying the set of six diluted DNA standards and diluted library samples via qPCR, using the KAPA SYBR^®^ FAST qPCR Master Mix and primers targeting the Illumina^®^ P5 and P7 flow cell oligo sequences. The qPCR was performed with the following cycling protocol: 95°C for 5 min, 35 cycles of 95°C for 30 s, 55°C for 30 s, and 60°C for 45 s. The average Cq score for each DNA standard was plotted against log_10_ of concentration (pM) to generate a standard curve. The concentrations of diluted library samples were then calculated against the standard curve, using absolute quantification. Final libraries were pooled in equimolar amounts, denatured and diluted to 4 pM before loading onto the MiSeq flow cell and sequenced on Illumina MiSeq platform (Illumina, San Diego, CA). According to Illumina protocol, 15% of PhiX Control library was combined with the amplicon library. MiSeq reagent Kit v3 (600 cycles) (Illumina, San Diego, CA) was used for library denaturating and for MiSeq sample loading. Sequencing was performed on Illumina MiSeq platform using a 2 × 300 bp paired end protocol.

#### Sequencing data analysis

The sequencing raw data were processed by the QIIME pipeline [50] using the “closed reference” out picking strategy. Raw reads quality has been checked using FastQC v0.11.2 [51], and R1 and R2 paired reads were joined using QIIME with the “SeqPrep” join method. The quality control was performed by QIIME, setting the phred_quality_threshold to 19 (Phred ≥ Q20). Reads were collected into OTUs (with identity ≥ 97%) using QIIME closed reference otu picking strategy against reference QIIME formatted Greengenes v.13.8 database (http://greengenes.lbl.gov). The taxonomical classification was performed down to genus level. OTUs assigned to the phylum *Cyanobacteria* (class *Chloroplast*), were considered potential plant contaminants and removed from the downstream analysis. Reads of mitochondrial or eukaryotic origin were also excluded. Singletons (OTUs with only one read associated) were excluded using the “filter_otus_from_otu_table.py" QIIME script.

Alpha and beta diversity statistics have been performed using QIIME scripts ‘alpha_rarefection.py’ and ‘jackknifed_beta_diversity_.py’, respectively. In the calculation of alpha diversity metrics, the normalization was performed using the "rarefaction" QIIME process with standard parameters setting the “max_rare_depth” (upper limit of rarefaction depths) to lowest sample size. Alpha diversity metrics were calculated using ‘observed species’, ‘Chao1 index’ (species richness estimator), ‘Shannon’s diversity index’ and ‘Good’s coverage’. An alpha-rarefaction plot was created for each metric. The alpha diversity values at the same rarefaction level (at the lowest sample size) were calculated.

Beta diversity metrics is an estimation of between-sample diversity of microbial profile and it was calculated by QIIME ‘jackknifed_beta_diversity_.py’ script. This script performed a jackknife iterative resampling method to normalize data, using a subsampling at 75% of the lowest sample size. We used both weighted (presence/absence/abundance matrix) and unweighted (presence/absence matrix) UniFrac distances [52,53]. The distance matrices were graphically visualized by three-dimensional PCoA representations.

#### Definition of the overall core community

Core microbiome analysis was performed in QIIME using the ‘compute_core_microbiome.py’ script. For this study the core microbiome was defined as the OTUs present in 80% of the samples regardless of diet.

### Statistical analysis

Normality and homoscedasticity of all data were checked by Shapiro–Wilk’s and Levene’s test, respectively, using STATISTICA v.7 (StatSoft, Inc). One-way analysis of variance (ANOVA) was performed on growth performance, feed conversion and α-diversity data. Statistical significance was set at *P*-value < 0.05, and Fisher's Least Significant Difference (LSD) test was applied for multiple comparisons, when the overall ANOVA resulted significant.

The number of reads across samples was normalized by sample size and the relative abundance (%) of each taxon was calculated. Only those taxa with an overall abundance of more than 1% (up to family level) and 0.5% at genus level were considered for statistical analysis.

Statistical analysis of intestinal microbial profiles was performed using the Statistical Analysis of Metagenomics Profiles (STAMP) program (http://kiwi.cs.dal.ca/Software/STAMP), retaining unclassified reads [54]. *P*-values were calculated by ANOVA followed by Tukey-Kramer post-hoc test and correction of multiple testing was done using Benjamini–Hochberg False Discovery Rate (FDR) method [55].

Differences in the beta diversity of bacterial communities were verified using the non-parametric Permutational Multivariate Analysis of Variance (PERMANOVA) and adonis tests with 999 permutations. Both tests were available with QIIME script ‘compare_categories.py’. A “by diet” pairwise significance test was also performed. For each pairwise contrast a filtered distance matrix containing only the samples to be compared was created using the “filter_distance_matrix.py” QIIME script, then a PERMANOVA significance test on each pairwise filtered matrix was performed using the “compare_categories.py” QIIME script.

## Results

### Growth and feed efficiency parameters of fish fed different diets

For the entire duration of the trial, mortality was negligible (< 1 percentage) and not correlated with a specific diet whereas final body weight data showed a diet effect (*P* < 0.05), revealing significant differences between experimental groups (Table 5). Indeed, at the end of the 12-week feeding trial, mean body weight of fish fed with diets E (293.78 ± 51.30 g) and G (298.28 ± 48.24 g) was significantly higher than the weight of other groups (*P* < 0.05), whereas fish fed diet F reached a mean body weight similar to fish fed diet E (control), but significantly lower than that of the group G (*P* < 0.05). Among all feeding groups, fish fed diet A showed the lowest mean mass value (251.77 ± 41.90). In line with weight data, the best SGR were observed in fish fed diets E, F, and G. Fish receiving diet A, B and C presented, in contrast, the lowest values (*P* < 0.05) (Table 5), whereas fish fed diet D showed an intermediate SGR value. Fish fed diets E, F, and G were better able to utilize energy for growth, too, as indicated by their lower FCR values (*P* < 0.05), which were 0.89, 0.91, and 0.89, respectively (Table 5). Trout fed diets A, B, and C showed, instead, the highest FCR values whereas fish fed diet D were positioned in between. Conversely, condition factor (K), that was calculated considering the entire experimental period (12 weeks), did not resulted significantly affected by diet (Table 5).

**Table 5:** Final mean body weight, specific growth rate (SGR), feed conversion ratio (FCR), and condition factor (K) values of trout fed with different diets. The final weight data represent mean value ± SD (n = 220 fish per diet). Different letters indicate statistically significant difference between groups (*P*<0.05).

Diet	Final weight (g)	SGR	FCR	K
**A**	251.77 ± 42.83^e^	1.19 ± 0.03^d^	1.06 ± 0.034^a^	1.14 ± 0.11
**B**	264.66 ± 46.92^d^	1.23 ± 0.01^cd^	1.03 ± 0.007^ab^	1.13 ± 0.13
**C**	264.80 ± 41.78^d^	1.25 ± 0.02^c^	1.01 ± 0.012^b^	1.14 ± 0.12
**D**	276.01 ± 44.30^c^	1.29 ± 0.01^b^	0.96 ± 0.003^c^	1.12 ± 0.11
**E**	293.57 ± 51.82^ab^	1.39 ± 0.02^a^	0.89 ± 0.007^d^	1.13 ± 0.13
**F**	286.12 ± 51.57^b^	1.35 ± 0.00^a^	0.91 ± 0.007^d^	1.12 ± 0.11
**G**	298.21 ± 48.67^a^	1.38 ± 0.02^a^	0.89 ± 0.013^d^	1.14 ± 0.14

### QIIME analysis of sequencing data

Sequencing data were exported as individual fastq files and has been deposited in European Nucleotide Archive (EBI ENA) under the accession code: PRJEB23230.

The sequence fastq files from the Illumina MiSeq were analysed using QIIME software. After filtering for quality, trimming length, and assigning taxonomies, the number of reads taxonomically classified according to the Greengenes database, discarding cyanobacteria and mitochondria reads, was 2,701,274. This value corresponded to an average number of 96,474 ± 68,056 reads per sample (range 5,573–283,511). We identified 5398 OTUs at 97% identity in trout faecal samples, of which 3304 were assigned to the genus level (S1 Dataset). After rarefaction, normalizing to the sample with the lowest number of sequences (5570 reads), the observed species number per sample was comprised between 113 and 682, corresponding to average counts per group comprised between 270 and 496 (Table 6). Good’s coverage values for all dietary groups were ≥ 0.96, indicating that sequencing coverage was attained and that the OTUs found in the samples were representative of the sampled population (Table 6). All the rarefaction curves, tended to plateau (S1 Fig). The number of observed species as well as the species richness index (Chao1) resulted not affected by diet type (Table 6). Similarly, Shannon’s diversity index, which accounts for both abundance and evenness of the species present, did not show significant differences between the tested feeding regimens. It reached, instead, a stable value in all samples, indicating that bacterial diversity in these communities was mostly covered (S1 Fig; Table 6).

**Table 6:** Number of reads per sample assigned to OTUs, and alpha diversity metrics values (normalized at the lowest sample size: 5570 sequences) of gut microbial community of trout fed with different diets for 12 weeks. Reported data are expressed as means ± SD (n = 4).

Diet	Reads	Observed species	Good’s coverage	Chao1	Shannon
A	92,418 ± 93,722	420 ± 67	0.97 ± 0.00	673 ± 95	5.65 ± 1.03
B	76,432 ± 70,693	270 ± 162	0.98 ± 0.01	540 ± 279	3.75 ± 2.00
C	104,521 ± 52,416	402 ± 102	0.97 ± 0.00	653 ± 129	5.28 ± 2.34
D	97,321 ± 68,096	494 ± 17	0.96 ± 0.01	803 ± 101	6.63 ± 0.12
E	155,045 ± 91,981	486 ± 91	0.97 ± 0.01	748 ± 188	6.67 ± 0.33
F	46,482 ± 15,417	496 ± 86	0.96 ± 0.01	767 ± 197	6.83 ± 0.14
G	103,097 ± 68,953	415 ± 250	0.96 ± 0.02	708 ± 348	4.49 ± 2.67
**Total number of reads taxonomically classified**			2,701,274		
**Mean number of reads/sample**			96,474 ± 68,056		
**Total number of OTUs**			5398		

### Faecal microbiome profiling of trout fed different diets

**Fig 1:**
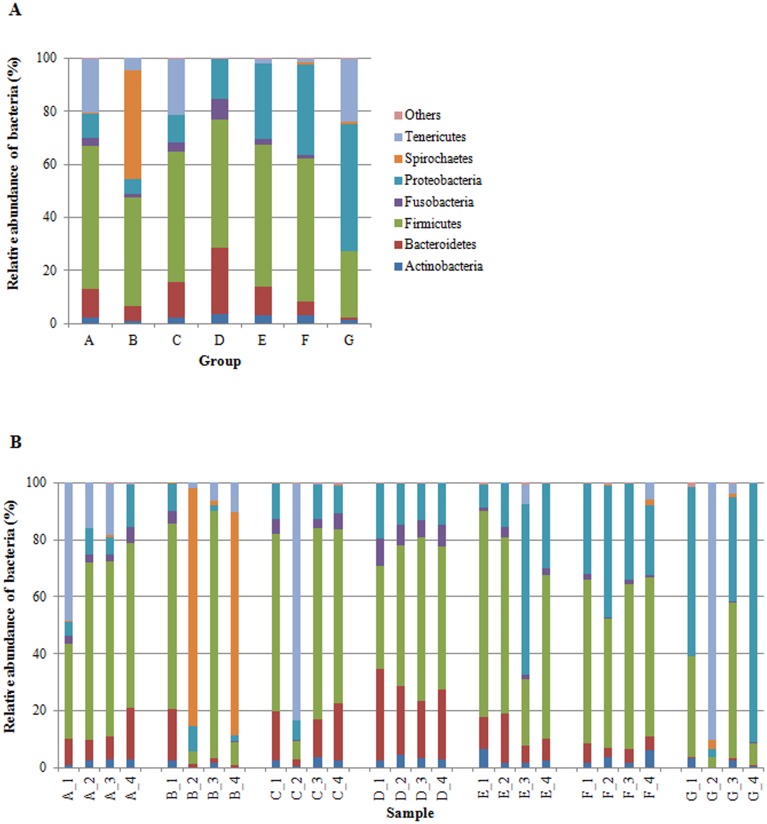
A, B. Relative abundance (%) of the overall most prevalent phyla in the different dietary groups (A) and in individual fish (B). In the figures, all bacteria with an overall abundance of ≥ 1% were reported. Bacteria with an abundance of ≤ 1% were pooled and indicated as “Others”.

**Fig 2:**
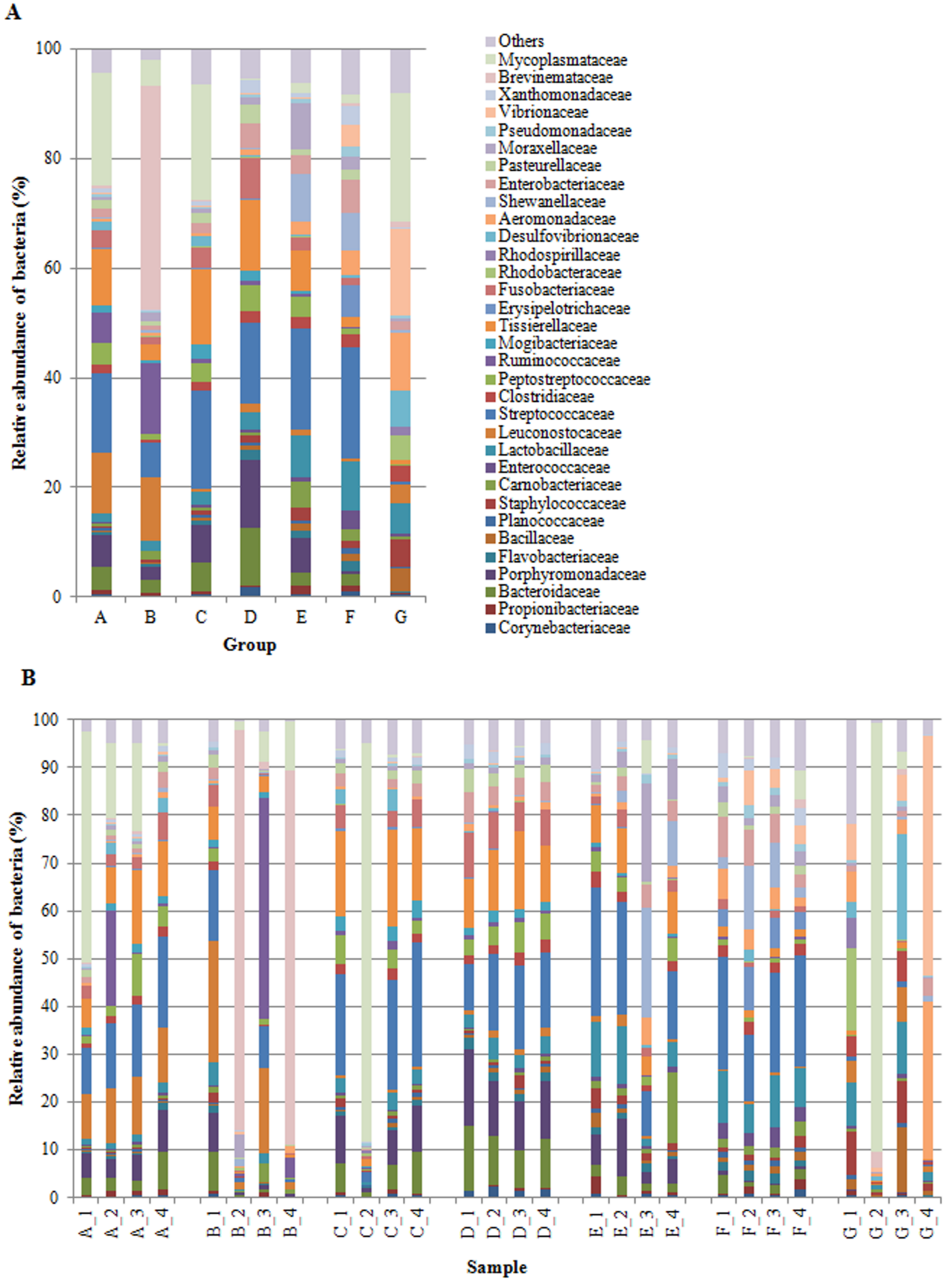
A, B. Relative abundance (%) of the overall most prevalent classes in the different dietary groups (A) and in individual fish (B). In the figures, all bacteria with an overall abundance of ≥ 1% were reported. Bacteria with an abundance of ≤ 1% were pooled and indicated as “Others”.

**Fig 3:**
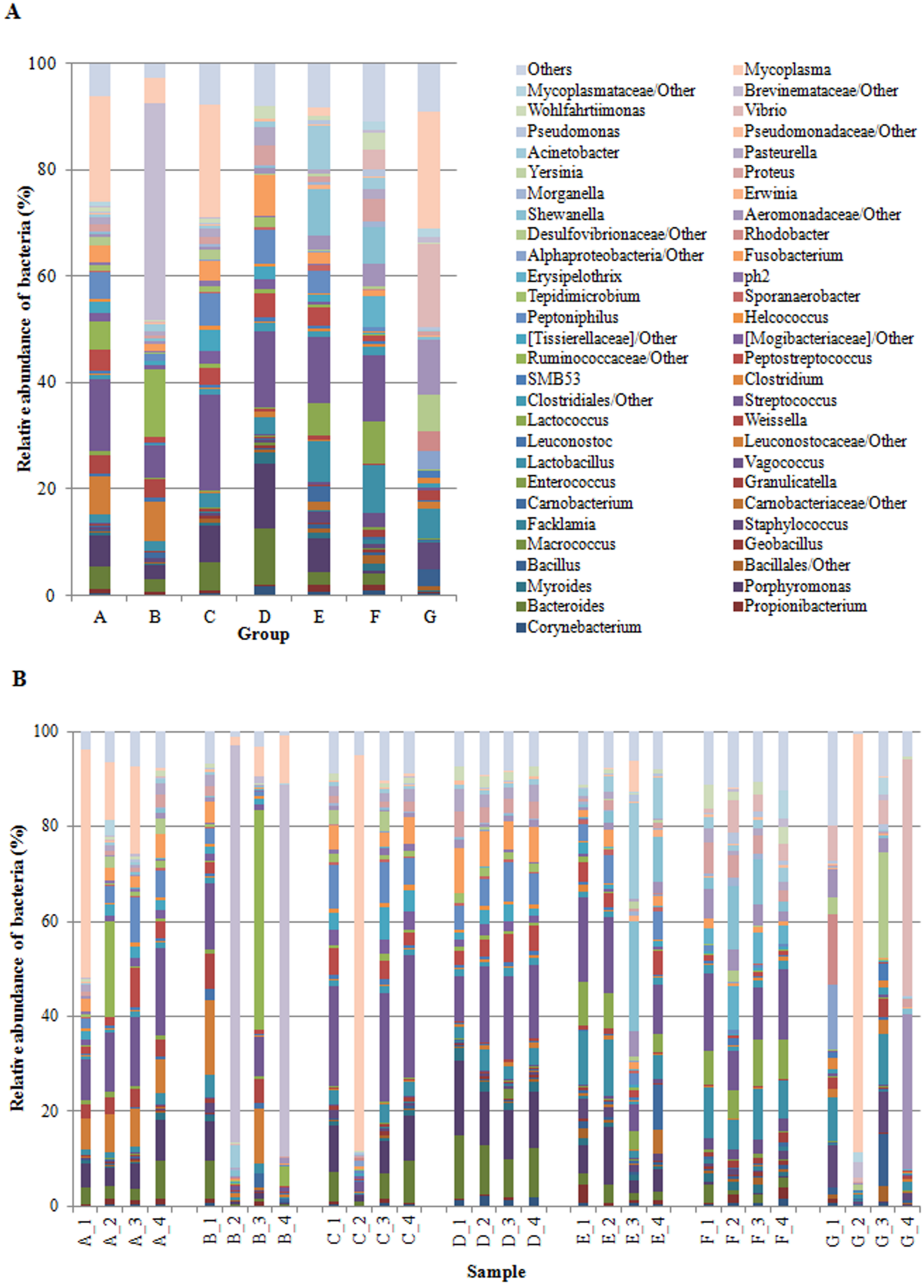
A, B. Relative abundance (%) of the overall most prevalent genera in the different dietary groups (A) and in individual fish (B). In the figures, all bacteria with an overall abundance of ≥ 0.5% were reported. Bacteria with an abundance of ≤ 0.5% were pooled and indicated as “Others”.

We successfully outlined the microbial community structures for each experimental group of fish at the phylum, class, order, family, and genus level. By considering only taxa with a relative abundance of more than 1% (up to family level), and more than 0.5% at genus level, the overall gut microbial community was mainly comprised of 7 phyla, 13 classes, 21 orders, 33 families and 41 genera. We have presented the profiles of intestinal microbial communities for each dietary group and individual fish at the phylum (Fig 1A and 1B), family (Fig 2A and 2B), and genus (Fig 3A and 3B) taxonomic level. In Table 7, is reported the relative abundance of all taxa that resulted significantly affected by diet. The result of post hoc multiple comparisons is shown in S1 Table. Irrespective of diet, the dominant phyla in our samples were *Firmicutes*, *Proteobacteria*, *Bacteroidetes*, and *Actinobacteria* (Fig 1A and 1B). A total of 211 OTUs constituted the core gut microbiota, i.e. OTUs that were shared by 80% of the samples irrespective of diet (S2 Fig, S2 Dataset). Among them, 42 OTUs were common to 100% of samples, showing a dominance of *Firmicutes* (28 OTUs) (S2 Dataset). Results of metagenomic analysis of trout faecal samples revealed that, at phylum level, *Fusobacteria* and *Bacteroidetes* were influenced by the diet. Indeed, in trout fed diet D the relative abundance of *Fusobacteria* was significantly higher than in other groups (*P* > 0.05) (Fig 1A). This was due to a significantly higher presence of bacteria assigned to *Fusobacteriaceae* family (7.58 ± 1.27%) of *Fusobacteriales* order (Fig 2A and 2B). Fish fed diets C and D had high amounts (*P* > 0.05) of bacteria belonging to *Bacteroidia* class (C: 12.28 ± 6.31%; D: 23.1 ± 4.04%). Specifically, trout fed these diets presented higher abundances of bacteria assigned to *Porphyromonadaceae* (C: 6.85 ± 3.53%; D: 12.46 ± 2.08%) and *Bacteroidaceae* (C: 5.33 ± 2.86%; D: 10.53 ± 1.98%) than other experimental groups (Fig 2A and 2B). A high percentage of bacteria belonging to *Bacteroidaceae* (from 2 to 4%) was also present in fish fed diets A, B, F, and E (control). Conversely, this bacterial family was practically absent in the gut of fish fed with diet G (0.03 ± 0.02). Similarly, *Porphyromonadaceae* family was scarcely represented (*P* < 0.05) in the gut of fish receiving diets F (0.48 ± 0.24%) and G (0.10 ± 0.10%) (Fig 2A and 2B). Several taxa belonging to *Firmicutes* and *Proteobacteria* phyla differed quantitatively between groups, thus resulting discriminatory for diet type. The *Enterococcaceae* family of the *Lactobacillales* order was significantly enriched (*P* < 0.001) in fish fed diet F (3.36 ± 0.48%) in comparison to other feeding groups (Fig 2A and 2B). Similarly, in the same dietary group, bacteria from *Erysipelotrichaceae* were more abundant (5.77 ± 2.30%) than in others (*P* < 0.001). Fish fed diets A, C, D, F, and control diet E showed a significantly higher amount of *Streptococcaceae* (from 14% to 20%) than fish of group G (0.6 ± 0.41%). Interestingly, the relative abundance of bacteria assigned to the [*Tissierellaceae*] family of the *Clostridia* class, was significantly higher in fish receiving diets with high content of plant proteins, i.e. diets A, C, and D (Table 1, Fig 2A and 2B), whereas bacteria corresponding to the *Clostridia* class were less abundant or almost absent in the gut of fish fed diets F (1.80 ± 0.23%) and G (0.58 ± 0.44%), which contained high levels of animal proteins, mainly PBM (Table 1).

**Table 7:** Mean relative abundance (%) ± SD of phyla, classes, orders, families and genera that were influenced by the diet. Statistical Analysis of Metagenomics Profiles (STAMP) software was used to test statistical significance between taxonomic groups abundances, unclassified reads were retained only for calculating frequency profiles. One-way ANOVA (*P* < 0.05), with an effect size (ETA-squared) and multiple test correction using the Benjamini-Hochberg FDR method, was applied followed by Tukey-Kramer post-hoc test. The result of post hoc multiple comparisons is reported in supplementary S1 Table.

Phylum	A	B	C	D	E	F	G	p-value (corr.)	Effect size
*Fusobacteria*	3.30 ± 1.27	1.34 ± 1.80	3.60 ± 1.93	7.59 ± 1.28	2.29 ± 0.92	1.30 ± 0.52	0.12 ± 0.12	3.8E-04	0.76
*Bacteroidetes*	10.75 ± 4.36	5.37 ± 7.17	13.15 ± 6.65	25.19 ± 4.37	10.65 ± 4.35	4.84 ± 1.34	0.53 ± 0.36	1.1E-03	0.71
**Class**									
*Erysipelotrichi*	0.24 ± 0.05	0.07 ± 0.06	0.38 ± 0.23	0.24 ± 0.08	0.20 ± 0.05	5.77 ± 2.30	0.07 ± 0.04	2.0E-05	0.83
*Flavobacteriia*	0.78 ± 0.45	0.42 ± 0.49	0.86 ± 0.37	2.08 ± 0.34	1.39 ± 0.39	1.99 ± 0.15	0.20 ± 0.12	1.0E-04	0.79
*Fusobacteriia*	3.30 ± 1.27	1.34 ± 1.80	3.60 ± 1.93	7.59 ± 1.28	2.29 ± 0.92	1.30 ± 0.52	0.12 ± 0.11	2.5E-04	0.76
*Bacteroidia*	9.94 ± 3.91	4.93 ± 6.66	12.28 ± 6.31	23.10 ± 4.04	9.05 ± 4.44	2.85 ± 1.34	0.21 ± 0.18	1.0E-03	0.71
**Order**									
*Erysipelotrichales*	0.24 ± 0.05	0.07 ± 0.06	0.38 ± 0.23	0.24 ± 0.08	0.20 ± 0.05	5.77 ± 2.30	0.07 ± 0.04	3.5E-05	0.83
*Flavobacteriales*	0.78 ± 0.45	0.42 ± 0.49	0.86 ± 0.37	2.08 ± 0.34	1.39 ± 0.39	1.99 ± 0.15	0.20 ± 0.12	1.8E-04	0.79
*Xanthomonadales*	0.82 ± 0.34	0.34 ± 0.43	1.02 ± 0.52	2.36 ± 0.47	0.74 ± 0.38	3.37 ± 1.15	0.11 ± 0.13	1.3E-04	0.79
*Fusobacteriales*	3.30 ± 1.27	1.34 ± 1.80	3.60 ± 1.93	7.59 ± 1.28	2.29 ± 0.92	1.30 ± 0.52	0.12 ± 0.11	3.4E-04	0.76
*Bacteroidales*	9.94 ± 3.91	4.93 ± 6.66	12.28 ± 6.31	23.10 ± 4.04	9.05 ± 4.44	2.85 ± 1.34	0.21 ± 0.18	1.5E-03	0.71
*Enterobacteriales*	1.61 ± 0.91	0.75 ± 0.98	1.82 ± 0.93	4.43 ± 1.10	3.41 ± 1.28	6.17 ± 2.16	1.58 ± 1.34	7.8E-03	0.65
*Pasteurellales*	1.42 ± 0.61	0.80 ± 1.12	1.81 ± 0.91	3.51 ± 0.96	0.88 ± 0.50	1.83 ± 0.72	0.03 ± 0.03	1.0E-02	0.64
**Family**									
*Enterococcaceae*	0.38 ± 0.17	0.12 ± 0.12	0.37 ± 0.19	0.46 ± 0.27	0.87 ± 0.27	3.36 ± 0.48	0.45 ± 0.31	6.6E-09	0.93
*Erysipelotrichaceae*	0.24 ± 0.05	0.07 ± 0.06	0.38 ± 0.23	0.24 ± 0.08	0.20 ± 0.05	5.77 ± 2.30	0.07 ± 0.04	3.5E-05	0.83
*Xanthomonadaceae*	0.82 ± 0.34	0.34 ± 0.43	1.02 ± 0.52	2.36 ± 0.47	0.74 ± 0.38	3.37 ± 1.14	0.11 ± 0.13	2.5E-04	0.79
*Fusobacteriaceae*	3.30 ± 1.27	1.33 ± 1.79	3.60 ± 1.93	7.58 ± 1.27	2.26 ± 0.93	1.28 ± 0.49	0.11 ± 0.11	6.3E-04	0.76
*Flavobacteriaceae*	0.69 ± 0.48	0.40 ± 0.48	0.78 ± 0.34	2.00 ± 0.34	1.25 ± 0.35	1.77 ± 0.36	0.14 ± 0.07	6.9E-04	0.75
*Aerococcaceae*	0.08 ± 0.03	0.03 ± 0.04	0.18 ± 0.10	0.13 ± 0.03	0.27 ± 0.27	0.85 ± 0.23	0.26 ± 0.14	8.0E-04	0.74
*Porphyromonadaceae*	5.82 ± 1.80	2.55 ± 3.29	6.85 ± 3.53	12.46 ± 2.08	6.52 ± 3.53	0.48 ± 0.24	0.10 ± 0.10	1.5E-03	0.72
*Corynebacteriaceae*	0.35 ± 0.16	0.27 ± 0.30	0.49 ± 0.27	1.71 ± 0.37	0.57 ± 0.23	0.87 ± 0.46	0.35 ± 0.26	2.7E-03	0.70
*Mogibacteriaceae*	1.50 ± 0.23	0.66 ± 0.62	2.54 ± 1.31	1.86 ± 0.39	0.53 ± 0.29	0.05 ± 0.03	0.01 ± 0.01	2.5E-03	0.70
*Bacteroidaceae*	4.07 ± 2.23	2.35 ± 3.33	5.33 ± 2.86	10.53 ± 1.98	2.38 ± 0.95	2.29 ± 1.16	0.03 ± 0.02	3.0E-03	0.69
[*Tissierellaceae*]	10.13 ± 3.60	2.79 ± 2.74	13.76 ± 7.18	12.75 ± 2.15	7.36 ± 2.12	1.80 ± 0.23	0.58 ± 0.54	3.6E-03	0.68
*Enterobacteriaceae*	1.61 ± 0.91	0.75 ± 0.98	1.82 ± 0.93	4.43 ± 1.10	3.41 ± 1.28	6.17 ± 2.16	1.58 ± 1.34	7.7E-03	0.65
*Pasteurellaceae*	1.42 ± 0.61	0.80 ± 1.12	1.81 ± 0.91	3.51 ± 0.96	0.88 ± 0.50	1.83 ± 0.72	0.03 ± 0.03	1.1E-02	0.64
*Lachnospiraceae*	0.42 ± 0.19	0.16 ± 0.20	0.58 ± 0.30	0.62 ± 0.14	0.19 ± 0.09	0.57 ± 0.14	0.03 ± 0.03	1.8E-02	0.61
*Streptococcaceae*	14.41 ± 3.43	6.28 ± 5.91	18.12 ± 9.42	14.71 ± 3.01	18.55 ± 7.09	20.42 ± 3.84	0.60 ± 0.41	2.2E-02	0.60
*Pseudomonadaceae*	0.50 ± 0.19	0.26 ± 0.26	0.41 ± 0.07	0.73 ± 0.10	0.95 ± 0.48	1.62 ± 0.62	0.55 ± 0.45	4.1E-02	0.57
**Genus**									
*Vagococcus*	0.20 ± 0.06	0.06 ± 0.07	0.20 ± 0.10	0.26 ± 0.14	0.51 ± 0.24	2.59 ± 0.35	0.13 ± 0.09	1.7E-10	0.96
*Lactococcus*	0.85 ± 0.18	0.32 ± 0.28	0.20 ± 0.10	0.18 ± 0.06	6.07 ± 2.43	7.79 ± 1.44	0.11 ± 0.08	1.4E-06	0.89
*Erysipelothrix*	0.12 ± 0.02	0.03 ± 0.03	0.17 ± 0.10	0.13 ± 0.07	0.17 ± 0.04	5.69 ± 2.32	0.05 ± 0.04	5.6E-05	0.83
*Sporanaerobacter*	0.31 ± 0.11	0.09 ± 0.10	0.36 ± 0.20	0.50 ± 0.06	0.98 ± 0.26	0.11 ± 0.11	0.02 ± 0.01	6.9E-05	0.82
*Tepidimicrobium*	0.98 ± 0.36	0.26 ± 0.31	1.01 ± 0.61	1.79 ± 0.16	0.07 ± 0.09	0.02 ± 0.02	0.14 ± 0.16	1.8E-04	0.80
*Ignatzschineria*	0.02 ± 0.01	0.00 ± 0.01	0.02 ± 0.01	0.06 ± 0.02	0.01 ± 0.01	0.00 ± 0.01	0.00 ± 0.00	4.10E-04	0.78
*Wohlfahrtiimonas*	0.76 ± 0.34	0.32 ± 0.40	0.93 ± 0.51	2.21 ± 0.42	0.67 ± 0.35	3.24 ± 1.17	0.09 ± 0.13	4.1E-04	0.78
*Fusobacterium*	3.22 ± 1.22	1.33 ± 1.79	3.56 ± 1.93	7.53 ± 1.27	2.15 ± 0.81	1.26 ± 0.48	0.03 ± 0.02	5.7E-04	0.77
*Granulicatella*	0.24 ± 0.10	0.23 ± 0.34	0.57 ± 0.32	0.27 ± 0.08	0.23 ± 0.10	1.42 ± 0.34	0.13 ± 0.18	8.1E-04	0.75
*Porphyromonas*	5.79 ± 1.78	2.54 ± 3.28	6.79 ± 3.50	12.37 ± 2.05	6.42 ± 3.51	0.41 ± 0.22	0.08 ± 0.08	2.2E-03	0.72
*Myroides*	0.67 ± 0.46	0.39 ± 0.46	0.72 ± 0.38	1.97 ± 0.34	1.07 ± 0.32	1.41 ± 0.39	0.07 ± 0.07	2.1E-03	0.72
*Proteus*	1.35 ± 0.77	0.57 ± 0.78	1.33 ± 0.67	3.68 ± 0.89	1.11 ± 0.52	4.36 ± 1.48	0.63 ± 0.71	2.2E-03	0.72
*Helcococcus*	0.51 ± 0.16	0.22 ± 0.20	0.77 ± 0.43	0.76 ± 0.09	0.17 ± 0.09	0.04 ± 0.05	0.00 ± 0.00	3.0E-03	0.71
*Corynebacterium*	0.35 ± 0.16	0.27 ± 0.30	0.49 ± 0.27	1.71 ± 0.37	0.57 ± 0.23	0.87 ± 0.46	0.35 ± 0.26	3.4E-03	0.70
*Bacteroides*	4.07 ± 2.23	2.35 ± 3.33	5.33 ± 2.86	10.53 ± 1.98	2.38 ± 0.95	2.18 ± 1.08	0.03 ± 0.02	4.0E-03	0.69
*pH2*	0.43 ± 0.10	0.09 ± 0.10	0.97 ± 0.56	0.24 ± 0.11	0.10 ± 0.11	0.01 ± 0.01	0.00 ± 0.00	8.8E-03	0.67
*Enterococcus*	0.12 ± 0.09	0.04 ± 0.04	0.10 ± 0.06	0.13 ± 0.09	0.25 ± 0.08	0.50 ± 0.19	0.21 ± 0.13	1.6E-02	0.64
*Pasteurella*	1.33 ± 0.56	0.71 ± 1.01	1.67 ± 0.85	3.25 ± 0.92	0.80 ± 0.51	1.72 ± 0.65	0.01 ± 0.01	1.6E-02	0.64
*Peptoniphilus*	5.20 ± 2.71	1.18 ± 1.28	5.97 ± 3.34	6.34 ± 1.26	4.42 ± 1.46	0.79 ± 0.17	0.08 ± 0.04	1.5E-02	0.64

Contrariwise, fish fed diets rich in PBM were characterized by a higher abundance of bacteria assigned to *Proteobacteria* phylum (Fig 1A and 1B). This phylum constituted 34.04 ± 7.76% and 47.44 ± 32.28% of the entire intestinal microbiome of trout fed with diets F and G respectively. Specifically, *Enterobacteriaceae*, *Xanthomonadaceae*, and *Pseudomonadaceae* families of the *γ*-*Proteobacteria* class were enriched in the intestine of fish fed diet F (Fig 2A and 2B).

At genus level (Fig 3A and 3B) the percentage of unassigned sequences was remarkable, in particular for fish of groups B (64.93%) and G (30.58%). Nevertheless, by considering the samples in their entirety, forty-one genera were identified. Of these, twenty-four genera belonged to *Firmicutes* phylum, eleven to *Proteobacteria*, three to *Bacteroidetes*, two to *Actinobacteria*, and only one genus belonged to *Actinobacteria* (Fig 3A and 3B). Among *Firmicutes*, the most abundant genera identified in all fish, except for those fed diet G, were *Streptococcus*, *Lactobacillus*, *Peptostreptococcus*, and *Peptoniphilus*. The latter, a member of *Clostridiales* order, was more abundant (*P* > 0.05) in faecal samples of trout receiving diets A (5.20 ± 2.71%), C (5.97 ± 3.34%), and D (6.34 ± 1.26%). Genus *Lactococcus* was enriched (*P* < 0.001) in fish fed with diets E (6.07 ± 2.43) and F (7.79 ± 1.44). Besides *Lactococcus*, other two genera of *Lactobacillales* order, i.e. *Vagococcus* (2.59 ± 0.35%) and *Enterococcus* (0.5 ± 0.19%), were more abundant in F than in other dietary groups. The *Proteobacteria* phylum was mainly represented by the genera *Proteus* and *Pasteurella* (Fig 3A and 3B), which were, together with *Wohlfahrtiimonas* genus, significantly affected by diet. In fish fed diet G, several *Proteobacteria* were identified in the gut, but they belonged to different genera such as: *Vibrio* (15.84%), unclassified *Aeromonadaceae* (10.53%), and *Rhodobacter* (3.79%) (Fig 3A and 3B). Bacteria from *Vibrio* genus were also found in faecal samples of diet F fed trout in which they represented about 4.0%. In addition, trout fed with diet E and F showed a high abundance of genus *Shewanella*, amounting to 8.77% and 6.77%, respectively (Fig 3A). Fish of group E also had a relatively high percentage of bacteria from *Acinetobacter* genus (Fig 3A and 3B). The phylum *Actinobacteria* was mainly represented by genera *Propionibacterium* and *Corynebacterium*. *Corynebacterium* genus resulted more abundant (*P* < 0.05) in fish fed diet D (1.71 ± 0.37) in comparison to other groups. Within *Bacteriodetes* phylum, *Bacteroides*, *Porphyromonas* and *Myroides* were the most abundant genera observed in our samples, and fish fed with diets C, D, and E generally showed the higher percentage (*P* < 0.05) of these genera in comparison to other groups (Table 7). Finally, *Mycoplasma* genus (Fig 3B) was identified in all samples, but in much lower quantities in fish fed with diets D, F and control diet E.

### Principal coordinate analysis (PCoA) of intestinal bacterial communities

**Fig 4:**
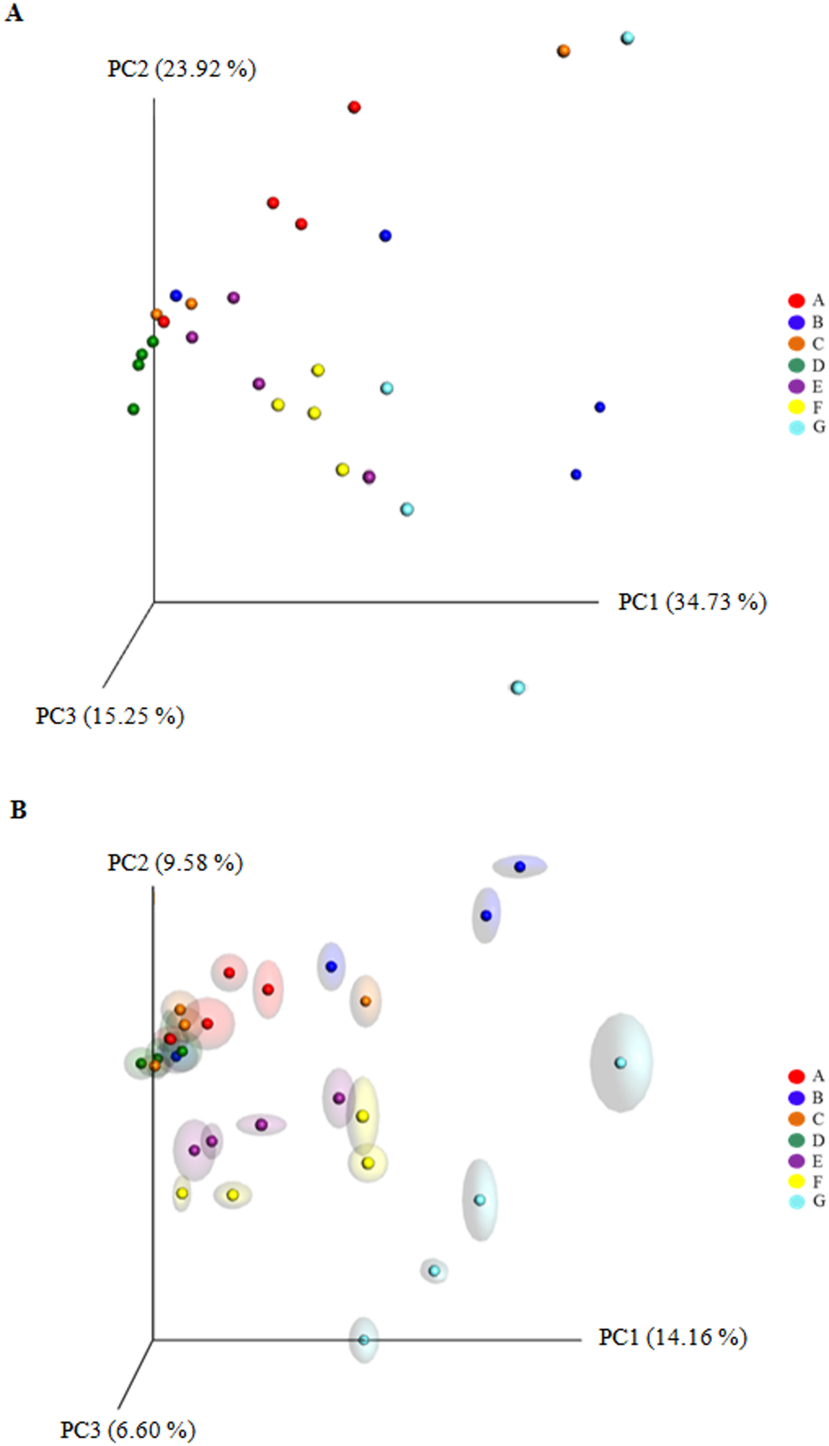
A, B. Beta diversity metrics. Principal coordinate analysis (PCoA) of Weighted (A) and Unweighted (B) Unifrac distances of gut microbial communities associated to different diet. The figures show the plot of individual fish (4 fish/diet) according to their microbial profile at genus level. Red = diet A; blue = diet B; orange = diet C; green = diet D; violet = control diet E; yellow = diet F; light blue = diet G.

QIIME pipeline was used to compute microbial beta diversity metrics; both weighted and unweighted UniFrac analyses were performed (Fig 4A and 4B). Data of UniFrac matrices were projected onto three-dimensional plots using principal coordinates analysis (PCoA). Weighted PCoA showed that most of samples were broadly indistinguishable and clustered together except for fish D and F which clustered according to diet (Fig 4A). Conversely, diet definitely affected unweighted UniFrac. Indeed, unweighted UniFrac PCoA revealed a clear clustering of samples by diet (Fig 4B). High animal-to-animal variation was observed in the group G, whose individual microbiomes appeared, indeed, to be more widely distributed on the first principal coordinate PC1 (14.16%).

The statistical analysis (permutation multivariate analysis PERMANOVA and Adonis test) totally reflected PCoA plots results, indicating a significant divergence between groups for both weighted (*P* = 0.002; R^2^ = 0.45; Pseudo-F = 2.82) and unweighted (*P* = 0.001; R^2^ = 0.33; Pseudo-F = 1.74) UniFrac distance matrices (Table 8). Pairwise test on the weighted UniFrac data showed that only fish fed diet D significantly diverged (*P* < 0.05) from all other groups, while fish fed with diet F was similar only to control group E (Table 8). Result of pairwise test on unweighted UniFrac data revealed, that samples clustered in three distinct groups, one constituted by A, B, C and D samples, one by F and control E, and the last by samples G (*P* < 0.05) (Table 8).

**Table 8:** Permutation multivariate analysis PERMANOVA and Adonis test on weighted and unweighted UniFrac data of intestinal microbiomes of trout fed with different experimental diets.

**Adonis analysis**	**Unweighted UniFrac**		**Weighted UniFrac**	
	*P*-value	R^2^	*P*-value	R^2^
	0.001	0.33	0.002	0.45
**PERMANOVA analysis**	**Unweighted UniFrac**		**Weighted UniFrac**	
	*P*-value	Pseudo-F	*P*-value	Pseudo- F
One-way				
Diet	0.001	1.74	0.001	2.82
**PERMANOVA Pairwise test**:				
A *vs* B	0.103		0.156	
A *vs* C	0.231		0.573	
A *vs* D	0.123		0.028	
A *vs* E	0.034		0.067	
A *vs* F	0.026		0.030	
A *vs* G	0.028		0.057	
B *vs* C	0.087		0.197	
B *vs* D	0.101		0.028	
B *vs* E	0.051		0.130	
B *vs* F	0.026		0.036	
B *vs* G	0.062		0.152	
C *vs* D	0.058		0.033	
C *vs* E	0.037		0.200	
C *vs* F	0.029		0.012	
C *vs* G	0.025		0.100	
D *vs* E	0.036		0.027	
D *vs* F	0.037		0.029	
D *vs* G	0.024		0.034	
E *vs* F	0.090		0.190	
E *vs* G	0.026		0.109	
F *vs* G	0.024		0.032	

## Discussion

Animal by-product meals from the rendering industry could be the most promising and suitable alternative to FM ingredients in aquaculture practice [19] due to their high content of essential amino acids and water-soluble proteins [16]. To date, several data are available on the effect of animal by-product meals on fish growth performances [16,17,27,45,56–58], but still very few studies have been conducted on their effect on fish gut microbiota [45,59,60]. Therefore, the information obtained in this study on the effects of substitution of FM with animal by-product meals on both, fish growth performance and intestinal microbiota biodiversity, represents a contribution to our knowledge.

During the feeding trial, trout survival rate was over 99% in all groups and no disease outbreaks occurred. Despite this, a clear diet effect was observed on fish growth. Indeed, trout fed commercial diets F and G grew as well as fish fed diet E (control) that was rich in FM (37.3%) and PBM free. The same fish displayed the best FCR and SGR values, too. These formulations were characterized by the highest content in animal proteins, of which PBM constituted the majority, whereas FM accounted for only 20% and 11%, respectively. Our findings are in agreement with previous studies conducted on rainbow trout [16,17,56,61] and other fish species, such as largemouth black bass (*Micropterus salmoides*) [62], hybrid striped bass (*Morone chrysops* x *M*. *saxatilis*) [57,58], cobia (*Rachycentron canadum*) [63] and Atlantic salmon (*Salmo salar*) [18]. In these species, good results in terms of growth rate were reported when PBM was used to replace FM in the diet. In particular, in Atlantic salmon, a test diet with 20% of FM replaced by PBM did not affect weight gain, feed intake and FCR with respect to a FM-based diet [18]. Similarly, in rainbow trout, a 30% replacement of FM with PBM yielded growth performances indexes comparable to those of fish fed with a FM-based control diet [61]. Burr and colleagues [16] reported that FM levels in rainbow trout feeds could be reduced to 10% without affecting fish growth if blends of animal and plant proteins are used. Always in trout [17], even a total substitution of FM with PBM, led to a high protein retention without apparent effects on growth. Contrariwise, Asian seabass (*Lates calcarifer*) fed a diet based on a mix of animal and plant proteins with FM inclusion rate reduced to 6%, showed a higher FCR value than control fish fed with a FM-based diet [45]. An increase in FCR was also found in Coho salmon (*Oncorhynchus kisutch*), when PBM was the primary protein-providing ingredient [64].

In terms of growth and feeding efficiency parameters, A and B definitely proved to be the worst formulations among all diets tested in the present feeding trial, followed by diet C. Better growth and feeding performances were obtained in fish receiving diet D, though their growth parameters were significantly lower than the control group E. The reduced growth rate observed in these trout was not due to an amino acid deficiency or imbalanced levels of essential amino acid (EAA). Indeed, despite varying proportion of different dietary protein sources, the amino acid profile was quite similar among experimental diets and the EAA levels exceeded the estimated EAA requirements for rainbow trout [15]. In fact, compared to control diet E, the content of EAA (such as Lys, Met and Thr) was 80% or above for all the diets. The amount and proportion of different fatty acids in the diet are important issues, too. In rainbow trout, it has been proved by time that n-3 fatty acids are essential for good growth and survival [65,66], and the substitution of FM with alternative terrestrial animal or plant proteins could alter the dietary n-3:n-6 fatty acid ratio. Actually, diets C and D, which were characterized by high vegetable and PBM inclusion, and low FM and fish oil content, showed clear differences in terms of fatty acids composition. In comparison to other formulations, C and D diets contained a higher amount of linoleic acid (18:2n-6), a lower content of eicosapentaenoic acid (EPA) (20:5n-3) and docosahexaenoic acid (DHA) (22:6n-3) and consequently, a lower n-3:n-6 fatty acid ratio. Although the n-3 LC-PUFA requirement for trout is low (0.4–0.5%) [15,67] and it was fulfilled by diets C and D, dietary levels of EPA + DHA, as well as those of linoleic acid could have affected fish growth performances. Indeed, reduced growth rates were observed in trout receiving diets containing high concentration of linoleic acid (18:2n-6) (5% of diet) [68], whereas in salmon, better performances were obtained with dietary EPA + DHA levels between 2.7 and 3.4% of total fatty acids [69] and concentrations of linoleic acid (18:2n-6) lower than 1% [64]. However, beyond the AA and FA profiles, several other factors could be responsible of the reduced fish growth including nutrient digestibility and antinutritional factors. In this regard, a recent study reported that salmon fed a diet with a mix of soy protein concentrate (30%) and poultry meal (6%) or a diet with 58% poultry meal showed reduced apparent digestibility of crude protein, amino acids, and lipids as compared to fish receiving FM-based diet [70]. Moreover, the same study showed that certain plant protein ingredients, such as soybean meal and soy protein concentrate, increased faecal water content in the distal intestine creating a diarrhoea-like condition that impaired gut function and reduced fish growth.

Even more interesting were the results obtained from our metagenomic analysis. Up to date, several studies have used cutting edge technologies, such as NGS, to evaluate the effect of substitution of FM with plant proteins on fish intestinal microbiota [43,44,71–73]. However, to the best of our knowledge, the present study is one of the very few researches to have investigated the effects of a diet with alternative terrestrial animal protein sources on fish gut microbiome [45,59,60] and the first one in rainbow trout. We analysed trout intestinal microbiome was by means of Illumina MiSeq sequencing of 16S rRNA gene. Fish used in our research were all female, obtained from a single supplier and grown under the same environmental conditions of an aquaculture facility, thereby limiting the variations due to environment and sex. Although diet is one of the main factors affecting the intestinal microbial composition of vertebrates, including fish, gut microbiota is also affected by fish developmental stage, gender, and farming conditions [28,74,75].

In line with previous studies on rainbow trout, our results indicated that gut microbiota of this species was dominated by *Firmicutes*, *Proteobacteria*, *Bacteroidetes* and *Actinobacteria* taxa. These phyla usually constitute the “core gut microbiota” of rainbow trout regardless of the diet type [37,38,43,44,71,74]. Actually, *Proteobacteria*, *Firmicutes*, and *Bacteroidetes* represent up to 90% of fish intestinal microbiota in different marine and freshwater species [40,45,76,77]. The presence of similar bacterial taxa in the gut microbiota of multiple fish species indicates that these bacteria are involved in important host gut functions, such as digestion, nutrient absorption, and immune response [37]. However, recent studies reported that *Tenericutes* were the prominent phylum, being *Mycoplasma* the dominant genus in the distal intestinal microbiome of rainbow trout [73,78]. In our study, *Mycoplasma* was detected in all samples, but the quantification was often several magnitudes lower than the other genera examined. As suggested by Harviksen *et al*. [60] it may be due to the difficulty in extracting DNA from bacteria with no cell wall.

The number of reads per sample did not differ between groups and no overall effect on bacterial richness and diversity was observed in response FM substitution with different protein blends. Similarly, replacing FM with a mix of terrestrial animal and plant proteins did not induce significant changes in gut microbial richness, alpha diversity indices, and observed number of species in Asian seabass (*Lates calcarifer*) [45]. In salmon, instead, the observed species parameter of alpha diversity metric presented higher value in fish fed poultry meal-based diet than in fish fed a control FM-based diet, whereas, in agreement with our study, Shannon’s diversity index did not show significant differences between dietary groups [59]. The lack of an effect on bacterial diversity should be considered as a positive result since the reduction in diversity may provide less competition for opportunistic or invading pathogens, which could thus easily colonize the gastrointestinal tract of fish [45].

Although all the rainbow trout used in this nutritional study showed similar intestinal bacterial communities, the relative abundance of several taxa displayed a significant statistical interaction with the diet. Both weight and unweighted UniFrac PCoA of bacterial communities revealed a relationship between diet type and microbiota associated to fish intestine, showing clustering of samples by diet, especially in the PCoA plot of the unweighted UniFrac data. However, some groups showed greater dispersion than others did. This was an expected result given that large individual variations even between fish of similar genetic background fed with the same diet and maintained under the same environmental conditions, has been described in previous reports [42,44,79].

Several studies have demonstrated the impact of marine versus terrestrial plant-derived ingredients on gut microbiota of rainbow trout [11,38,43,44]. These studies revealed that plant ingredients in the diet were often associated with a higher *Firmicutes*:*Proteobacteria* ratio in comparison to FM-based diet, which favoured instead, the presence of *Proteobacteria*. The inclusion of at least 25% of plant proteins in the diet of our fish favoured the presence of genera from the *Firmicutes* phylum regardless of the content level of animal proteins. Conversely, gut microbiota of fish fed diet G, with the lowest plant protein percentage (20%) and the highest content of animal proteins (80%), was found to be rich in *γ-Proteobacteria*. Similarly, previous studies in trout reported that the presence of *Proteobacteria* was favoured by an animal protein-based diet [38,43,44]. Different genera of lactic acid bacteria such as *Streptococcus*, *Lactobacillus*, *Leuconostoc*, and *Carnobacterium* belonging to *Firmicutes*, constitute a normal part of the intestinal microbiota of fish and are generally considered beneficial microorganisms associated with a healthy intestinal epithelium [80,81]. These bacterial genera, indeed, were used as probiotics for fish as well as for other vertebrates [82–85]. Several genera belonging to *Lactobacillales* and *Clostridiales*, orders were significantly affected by feeding formulations tested in our study. This was in line with recent literature data reporting that, although the microbiota composition of cultured rainbow trout was very resistant to diet changes, dietary variations were associated with changes in the relative abundance of *Lactobacillaceae*, *Streptococcaceae*, *Staphylococcaceae*, and *Clostridiales* [71]. In particular, the relative abundance of bacteria belonging to *Streptococcaceae*, *Enterococcaceae*, *[Tissierellaceae]*, and *Carnobacteriaceae* families varied between our feeding groups. *Lactobacillales* order was highly represented in the intestine of trout fed diet E (control) and F as well as in fish fed diets A, C, and D. Conversely, bacteria belonging to this order were present, to a lesser extent, in faecal samples of fish fed with diets B and G. Similarly, digesta (faecal) samples of Atlantic salmon fed a diet containing soy protein concentrate (30%) and poultry meal (6%), as partial replacements of FM, presented significantly higher abundance of *Lactobacillales* genera *Streptococccus*, *Carnobacterium*, and *Lactococcus* [59]. In the same study, in accordance with our results, fish fed with a high percentage of poultry meal (58%) showed higher abundance of *γ-Proteobacteria*. In a previous study, in salmon, PBM inclusion led instead to a significant increase of *Corynebacteriaceae* and a significant decrease of *β-Proteobacteria*, *Bacilli*-like, *Streptococcaceae*, and *Peptostreptococcacea* in allochthonous bacterial community in comparison to a FM-based control group, whereas in autochthonous community, dietary PBM caused an increase in *Corynebacteriaceae* and *Streptococcaceae* [60]. In our study, we found a significant enrichment of *Corynebacteriaceae* family, represented by genus *Corynebacterium*, only in trout fed with diet D. It is interestingly to note that intestinal microbiome of group B, which showed the worst performances in terms of growth and feeding efficiency, was characterized not only by scarce amount of lactic acid bacteria but also by low abundance of bacteria assigned to *Clostridiales*. These differences could partly explain the poor growth performances observed in this fish group. In European sea bass (*Dicentrarchus labrax*), for example, changes in the composition of cecal microbiota deeply influenced weight gain, suggesting the involvement of bacterial community in energy harvesting from feed [86]. Actually, members of *Streptococcaceae*, *Lactobacillaceae*, *Enterobacteriacea* and [*Tissierellaceae*] families include several bacterial species that participate to anaerobic degradation of complex carbohydrates. The end products of such degradation are short chain fatty acids (SCFAs), which are then readily absorbed by the host thus contributing to the more efficient food energy utilization [87–90].

*Fusobacterium* genus was enriched in the intestine of trout fed diet D in comparison to all other dietary groups. It is known that bacteria belonging to *Fusobacteria* phylum can excrete butyrate [91] and synthesize vitamins [81]. Among the SCFAs, butyrate is considered the most important due to its numerous positive and well-documented effects on the health of intestinal tract and peripheral tissues in vertebrates [87,92–94]. Butyrate has, indeed, anti-inflammatory properties and the potential to stimulate the immune system [95–99]. For these reasons, we hypothesized that the intestinal presence of *Fusobacterium* could exert a beneficial effect on fish health. Actually, trout fed on diet D grew well and showed good feed efficiency parameters, although FM content in their diet was only at 11%. Therefore, a positive effect due to their gut microbiota composition could be reasonably hypothesised. These data represent a contribution if we consider that up to date, no other studies have established which are the microbial taxa that play a dominant role in SCFAs production in fish. Moreover, if we limit the comparison between gut microbiota only to groups G and F (trout fed the two formulations with the highest percentage of animal by-product meals), an adequate number (above 0.5%) of bacterial genera assigned to *Carnobacteriaceae*, *Streptococcaceae*, and *Enterococcaceae* families were found only in trout fed with diet F. Unweighted UniFrac PCoA analysis clearly showed that intestinal microbiome profile of fish fed diet F was more similar to that of control fish (diet E) than to other groups. This is a promising and encouraging result toward the use of animal by-product meals in aquaculture. On the other hand, although the severely reduced amount of *Lactobacillales* in fish fed diet G did not negatively affect SGR and FCR, it could have influenced the susceptibility to pathogens or opportunistic bacterial species. Indeed, microbiota of this group was dominated by *γ-Proteobacteria*, mainly represented by members belonging to *Aeromonadales* and *Vibrionales* orders, which include potential pathogen genera, such as *Photobacterium* and *Aeromonas*. Furthermore, the presence in the same fish group of an imbalanced microbiota, in which *Proteobacteria* phylum represented the dominant clade, could alter immune regulatory functions of the gut and contribute to development of diseases [100].

## Conclusions

In summary, taken together, our data revealed that animal by-product meals, particularly PBM, could be a valid alternative protein source for aquafeed production. These ingredients do not negatively affect fish growth performances, but rather could reduce the negative impacts of high inclusion rates of dietary plant proteins on fish growth. Adding PBM to trout diet introduced no changes in the total microbial diversity or richness. Changes to the intestinal microbiome composition that we found were actually due to the ratio between vegetable and animal proteins regardless of the animal proteins sources. In particular, intestinal abundance of specific taxa belonging to *Firmicutes* and *Proteobacteria* was discriminatory for diet type in trout. Among tested diets, formulation D provided the best results in terms of percentage of FM replacement, growth performance, and intestinal microbiota composition, whereas experimental feed B and commercial feed G had an adverse effect on the gut microbial community by reducing the abundance of *Lactobacillales*. By manipulating fish diet, it is possible to obtain positive effects on the composition of gut microbiota and, hence, on the host’s physiology. However, further experiments are needed to elucidate which are the feed ingredients that have the highest impact on the gut microbiota changes.

## Supporting information

S1 Fig Alpha diversity metrics. Rarefaction curves of faecal microbial communities from trout fed different diets. (**A**) Observed species, (**B**) species richness (Chao1), (**C**) Shannon’s diversity index. Data points represent the mean values (n = 4).
(TIF)

S2 Fig The common core microbiota. The *x*-axis represents the percentage of prevalence in all samples (n = 28) regardless of the diet type, the *y*-axis represents the number of shared OTUs.
(TIF)

S1 Table Result of Tukey-Kramer post-hoc test on relative abundance data of phyla, classes, orders, families and genera that were influenced by the diet. Significance codes: *P < 0.05, **P < 0.01, ***P < 0.001.
(DOCX)

S1 Dataset OTU table generated by QIIME pipeline. (XLSX)

S2 Dataset Core microbiota, list of shared OTUs at 80% and 100%. (XLSX)

## References

[1] Shepherd CJ, Jackson AJ (2013). Global fishmeal and fish-oil supply: inputs, outputs and Markets. Journal of Fish Biology 83:1046–1066. doi: 10.1111/jfb.12224 24090562 10.1111/jfb.12224

[2] Kristofersson D, Anderson JL (2006). Is there a relationship between fisheries and farming? Interdependence of fisheries, animal production and aquaculture. Marine Policy 30:721–725.

[3] Tacon AGJ, Metian M (2008). Global overview on the use of fish meal and fish oil in industrially compounded aquafeeds: trends and future prospects. Aquaculture 285:146–158.

[4] Tacon AGJ (2004). Use of fish meal and fish oil in aquaculture: a global perspective. Aquatic Resources Culture and Development 1:3–14.

[5] Pike I (2005). Eco-efficiency in aquaculture: global catch of wild fish used in aquaculture. International Aquafeed 8:38–40.

[6] Barlow S (2000). Fishmeal and fish oil: sustainable feed ingredients for aquafeeds. Global Aquaculture Advocate 4:85–88.

[7] Gatlin DM, Barrows FT, Brown P, Dabrowski K, Gaylord TG, Hardy RW, et al (2007). Expanding the utilization of sustainable plant products in aquafeeds: a review. Aquaculture Research 38:551–579.

[8] Francis G, Makkar HPS, Becker K (2001). Anti-nutritional factors present in plant derived alternate fish feed ingredients and their effects in fish. Aquaculture 199:197–227.

[9] Morris PC, Gallimore P, Handley J, Hide G, Haughton P, Black A (2005). Full-fat soya for rainbow trout (*Oncorhynchus mykiss*) in freshwater: effects on performance, composition and flesh fatty acid profile in absence of hind-gut enteritis. Aquaculture 248:147–161.

[10] Krogdahl Å, Penn M, Thorsen J, Refstie S, Bakke AM (2010). Important antinutrients in plant feedstuffs for aquaculture: an update on recent findings regarding responses in salmonids. Aquaculture Research, 41:333–344.

[11] Heikkinen J, Vielma J, Kemiläinen O, Tiirola M, Eskelinen P, Kiuru T, et al (2006). Effects of soybean meal based diet on growth performance, gut histopathology and intestinal microbiota of juvenile rainbow trout (*Oncorhynchus mykiss*). Aquaculture 261:259–268

[12] Santigosa E, García-Meilań I, Valentín MV, Navarro I, Pérez-Sańchez J, Gallardo MA (2011). Plant oils’ inclusion in high fish meal-substituted diets: effect on digestion and nutrient absorption in gilthead sea bream (*Sparus aurata* L.). Aquaculture Research 42:962–974.

[13] Zhang J-X, Guo L-Y, Feng L, Jiang W-D, Kuang S-Y, Liu Y, et al (2013). Soybean b-Conglycinin Induces Inflammation and Oxidation and Causes Dysfunction of Intestinal Digestion and Absorption in Fish. PLoS ONE 8(3):e58115 doi: 10.1371/journal.pone.0058115 23520488 10.1371/journal.pone.0058115 PMC3592885

[14] Penn MH, Bendiksen EÅ, Campbell P, Krogdahl Å (2011). High level of dietary pea protein concentrate induces enteropathy in Atlantic salmon (*Salmo salar* L.). Aquaculture 310:267–273.

[15] National Research Council (2011). Nutrient Requirements of Fish and Shrimp. National Academies Press, Washington, DC.

[16] Burr GS, Wolters WR, Barrows FT, Hardy RW (2012). Replacing fishmeal with blends of alternative proteins on growth performance of rainbow trout (*Oncorhynchus mykiss*), and early or late stage juvenile Atlantic salmon (*Salmo salar*). Aquaculture 334:110–116

[17] Badillo D, Herzka SZ, Viana MT (2014). Protein Retention Assessment of Four Levels of Poultry By-Product Substitution of Fishmeal in Rainbow Trout (*Oncorhynchus mykiss*) Diets Using Stable Isotopes of Nitrogen (δ15N) as Natural Tracers. PLoS ONE 9(9):e107523 doi: 10.1371/journal.pone.0107523 25226392 10.1371/journal.pone.0107523 PMC4166461

[18] Hartviksen M, Bakke AM, Vecino JG, Ringø E, Krogdahl Å (2014). Evaluation of the effect of commercially available plant and animal protein sources in diets for Atlantic salmon (Salmo salar L.): digestive and metabolic investigations. Fish Physiology and Biochemistry 40:1621–1637. doi: 10.1007/s10695-014-9953-4 24962539 10.1007/s10695-014-9953-4

[19] Davies SJ, Gouveia A, Laporte J, Woodgate SL, Nates S (2009). Nutrient digestibility profile of premium (category III grade) animal protein by-products for temperate marine fish species (European sea bass, gilthead sea bream and turbot). Aquaculture Research 40:1759–1769.

[20] Dong C, He G, Mai K, Zhou H, Xu W (2016). Palatability of Water-Soluble Extracts of Protein Sources and Replacement of Fishmeal by a Selected Mixture of Protein Sources for Juvenile Turbot (*Scophthalmus maximus*). Journal of Ocean University of China (Oceanic and Coastal Sea Research) 15(3):561–567.

[21] EC (2013). Commission Recommendation No 2013/165/EU of 27 March 2013 on the presence of T-2 and HT-2 toxin in cereals and cereal products. Official Journal of European Union L 91:12–15.

[22] Yu Y (2007). Replacement of fish meal with poultry byproduct meal and hydrolyzed feather meal in feeds for finfish In: Alternative Protein Sources in Aquaculture Diets (Lim C., Webster C.D. and Lee C.S. (Eds), The Haworth Press, New York, 51–93 pp.

[23] Cheng ZJ, Hardy WR (2002). Apparent digestibility coefficients of nutrients and nutritional value of poultry byproduct meals for rainbow trout *Oncorhynchus mykiss* measured in vivo using settlement. Journal of World Aquaculture Society 33:458–465.

[24] Choi WM, Lam CL, Mo WY, Wong MH (2015). The use of food wastes as feed ingredients for culturing grass carp (*Ctenopharyngodon idellus*) in Hong Kong. Environmental Science and Pollution Research 23(8):7178–85. doi: 10.1007/s11356-015-5465-8 26432269 10.1007/s11356-015-5465-8

[25] Hernández C, Olvera-Novoa MA, Hardy RW, Hermosillo A, Reyes C, González B (2010). Complete replacement of fishmeal by porcine and poultry by-product meals in practical diets for fingerling Nile tilapia *Oreochromis niloticus*: digestibility and growth performance. Aquaculture Nutrition 16:44–53.

[26] Barreto-Curiel F, Parés-Sierra G, Correa-Reyes G, Durazo-Beltrán E, Viana MT (2016). Total and partial fishmeal substitution by poultry by-product meal (petfood grade) and enrichment with acid fish silage in aquafeeds for juveniles of rainbow trout *Oncorhynchus mykiss*. Latin American Journal of Aquatic Research 44(2):327–335

[27] Pares-Sierra G, Durazo E, Ponce MA, Badillo D, Correa-Reyes G, Viana MT (2014). Partial to total replacement of fishmeal by poultry by-product meal in diets for juvenile rainbow trout (*Oncorhynchus mykiss*) and their effect on fatty acids from muscle tissue and the time required to retrieve the effect. Aquaculture Research 45:1459–1469.

[28] Nayak SK (2010). Role of gastrointestinal microbiota in fish. Aquaculture Research 41:1553–1573.

[29] David LA, Maurice CF, Carmody RN, Gootenberg DB, Button JE, Wolfe BE, et al (2014). Diet rapidly and reproducibly alters the human gut microbiome. Nature 505(7484):559–63. doi: 10.1038/nature12820 24336217 10.1038/nature12820 PMC3957428

[30] Ley RE, Hamady M, Lozupone C, Turnbaugh PJ, Ramey RR, Bircher JS, et al (2008). Evolution of mammals and their gut microbes. Science 320:1647–1651. doi: 10.1126/science.1155725 18497261 10.1126/science.1155725 PMC2649005

[31] Zoetendal EG, de Vos WM (2014). Effect of diet on the intestinal microbiota and its activity. Current Opinion in Gastroenterology 30:189–195. doi: 10.1097/MOG.0000000000000048 24457346 10.1097/MOG.0000000000000048

[32] Rawls JF, Samuel BS, Gordon JI (2004). Gnotobiotic zebrafish reveal evolutionarily conserved responses to the gut microbiota. Proceedings of the National Academy of Sciences USA 101(13):4596–4601. 10.1073/pnas.0400706101 PMC384792 15070763

[33] Rawls JF, Mahowald MA, Ley RE, Gordon JI (2006). Reciprocal gut microbiota transplants from zebrafish and mice to germ-free recipients reveal host habitat selection. Cell 127:423–433. doi: 10.1016/j.cell.2006.08.043 17055441 10.1016/j.cell.2006.08.043 PMC4839475

[34] Gómez GD, Balcázar JL (2008). A review on the interactions between gut microbiota and innate immunity of fish. FEMS Immunology and Medical Microbiology 52:145–154 doi: 10.1111/j.1574-695X.2007.00343.x 18081845 10.1111/j.1574-695X.2007.00343.x

[35] Llewellyn MS, Boutin S, Hoseinifar SH, Derome N (2014). Teleost microbiomes: the state of the art in their characterization, manipulation and importance in aquaculture and fisheries. Frontiers in Microbiology 5:207 doi: 10.3389/fmicb.2014.00207 24917852 10.3389/fmicb.2014.00207 PMC4040438

[36] Maslowski KM, Mackay CR (2010). Diet, gut microbiota and immune responses. Nature Immunology 12:5–9. 10.1038/ni0111-5 21169997

[37] Ghanbari M, Kneifel W, Domig KJ (2015). A new view of the fish gut microbiome: Advances from next-generation sequencing. Aquaculture 448:464–475

[38] Ingerslev H-C, Strube ML, von Gersdorff Jørgensen L, Dalsgaard I, Boye M, Madsen L (2014a). Diet type dictates the gut microbiota and the immune response against *Yersinia ruckeri* in rainbow trout (*Oncorhynchus mykiss*). Fish & Shellfish Immunology 40:624–633 25150450 10.1016/j.fsi.2014.08.021

[39] Araújo C, Muñoz-Atienza E, Nahuelquín Y, Poeta P, Igrejas G, Hernández PE, et al (2015). Inhibition of fish pathogens by the microbiota from rainbow trout (*Oncorhynchus mykiss*, Walbaum) and rearing environment. Anaerobe 32:7–14. doi: 10.1016/j.anaerobe.2014.11.001 25464142 10.1016/j.anaerobe.2014.11.001

[40] Ringø E, Zhou Z, Vecino JLG, Wadsworth S, Romero J, Krogdahl Å, et al (2016). Effect of dietary components on the gut microbiota of aquatic animals. A never-ending story? Aquaculture Nutrition 22:219–282.

[41] Batista S, Ozório ROA, Kollias S, Dhanasiri AK, Lokeshc J, Kironc V, et al (2016). Changes in intestinal microbiota, immune- and stress-related transcript levels in Senegalese sole (*Solea senegalensis*) fed plant ingredient diets intercropped with probiotics or immunostimulants. Aquaculture 458:149–157.

[42] Estruch G, Collado MC, Peñaranda DS, Tomás Vidal A, Jover Cerdá M, Pérez Martínez G, et al (2015). Impact of fishmeal replacement in diets for gilthead sea bream (*Sparus aurata*) on the gastrointestinal microbiota determined by pyrosequencing the 16S rRNA Gene. PLoS ONE 10(8):e0136389 doi: 10.1371/journal.pone.0136389 26317431 10.1371/journal.pone.0136389 PMC4552794

[43] Ingerslev H-C, von Gersdorff Jørgensen L, Strube ML, Larsen N, Dalsgaard I, Boye M, et al (2014b). The development of the gut microbiota in rainbow trout (*Oncorhynchus mykiss*) is affected by first feeding and diet type. Aquaculture 424–425:24–34.

[44] Desai AR, Links MG, Collins SA, Mansfield GS, Drew MD, Van Kessel AG, et al (2012). Effects of plant-based diets on the distal gut microbiome of rainbow trout (*Oncorhynchus mykiss*). Aquaculture 350–353:134–142.

[45] Apper E, Weissman D, Respondek F, Guyonvarch A, Baron F, Boisot P, et al (2016). Hydrolysed wheat gluten as part of a diet based on animal and plant proteins supports good growth performance of Asian seabass (*Lates calcarifer*), without impairing intestinal morphology or microbiota. Aquaculture 453:40–48

[46] Liu HJ, Chang BY, Yan HW, Yu FH, Lu XX (1995). Determination of amino acids in food and feed by derivatization with 6-aminoquinolyl-N-hydroxysuccinimidyl carbamate and reversed phase liquid chromatographic separation. Journal of AOAC International 78:736–744.

[47] Folch J, Lees M, Sloane-Stanley GH (1957). A simple method for the isolation and purification of total lipids from animal tissues. Journal of Biological Chemistry 226:497–509 13428781

[48] Santha NC, Ackman RG (1990). Nervonic acid versus tricosanoic acid as internal standards in quantitative gas chromatographic analyses of fish oil longer-chain n-3 polyunsaturated fatty acid methyl esters. Journal of Chromatography 533:1–10. 2150519 10.1016/s0378-4347(00)82182-9

[49] Klindworth A, Pruesse E, Schweer T, Peplies J, Quast C, Horn M, et al (2013). Evaluation of general 16S ribosomal RNA gene PCR primers for classical and next-generation sequencing-based diversity studies. Nucleic Acids Research 41: No.1 10.1093/nar/gks808 PMC3592464 22933715

[50] Caporaso JG, Kuczynski J, Stombaugh J, Bittinger K, Bushman FD, Costello EK, et al (2010). QIIME allows analysis of high-throughput community sequencing data. Nature Methods 7:335–336 doi: 10.1038/nmeth.f.303 20383131 10.1038/nmeth.f.303 PMC3156573

[51] Andrews S (2010). FastQC: a quality control tool for high throughput sequence data. http://www.bioinformatics.babraham.ac.uk/projects/fastqc

[52] Lozupone C, Knight R (2005). UniFrac: a new phylogenetic method for comparing microbial communities. Applied and Environmental Microbiology 71:8228–8235. doi: 10.1128/AEM.71.12.8228-8235.2005 16332807 10.1128/AEM.71.12.8228-8235.2005 PMC1317376

[53] Lozupone CA, Hamady M, Kelley ST, Knight R (2007). Quantitative and qualitative β diversity measures lead to different insights into factors that structure microbial communities. Applied and Environmental Microbiology 73:1576–1585. doi: 10.1128/AEM.01996-06 17220268 10.1128/AEM.01996-06 PMC1828774

[54] Parks DH, Beiko RG. (2010). Identifying biologically relevant differences between metagenomic communities. Bioinformatics 26: 715–721. doi: 10.1093/bioinformatics/btq041 20130030 10.1093/bioinformatics/btq041

[55] Benjamini Y, Hochberg Y. (1995). Controlling the false discovery rate: a practical and powerful approach to multiple testing. Journal of the Royal Statistical Society 57: 289–300.

[56] Gaylord TG, Sealey WM, Barrows FT, Myrick CA Fornshell G (2017). Evaluation of ingredient combinations from differing origins (fishmeal, terrestrial animal and plants) and two different formulated nutrient targets on rainbow trout growth and production efficiency. Aquaculture Nutrition 23(6): 1319–1328.

[57] Rawles SD, Riche M, Gaylord TG, Webb J, Freeman DW, Davis M (2006). Evaluation of poultry by-product meal in commercial diets for hybrid striped bass (*Morone chrysops* ♀ × *M*. *saxatilis* ♂) in recirculated tank production. Aquaculture 259:377–389.

[58] Rawles SD, Gaylord TG, McEntire ME, Freeman DW (2009). Evaluation of poultry by-product meal in commercial diets for hybrid striped bass, *Morone chrysops* x *M*. *saxatilis* in pond production. Journal of World Aquaculture Society 40:141–156.

[59] Gajardo K, Jaramillo-Torres A, Kortner TM, Merrifield DL, Tinsley J, Bakke AM, Krogdahl Å (2017). Alternative protein sources in the diet modulate microbiota and functionality in the distal intestine of Atlantic salmon (*Salmo salar*). Applied and Environmental Microbiology 83(5): e02615–16. doi: 10.1128/AEM.02615-16 27986728 10.1128/AEM.02615-16 PMC5311410

[60] Hartviksen M, Vecino JLG, Ringø E, Bakke AM, Wadsworth S, Krogdahl Å, Ruohonen K, Kettunen A (2014). Alternative dietary protein sources for Atlantic salmon (*Salmo salar* L.) effect on intestinal microbiota, intestinal and liver histology and growth. Aquaculture Nutrition 20:381–398.

[61] EL-Haroun ER, Azevedo PA, Bureau DP (2009). High dietary incorporation levels of rendered animal protein ingredients on performance of rainbow trout *Oncorhynchus mykiss* (Walbaum, 1972). Aquaculture 290:269–274

[62] Subhadra B, Lochman R, Rawles S, Chen R (2006). Effect of fish-meal replacement whit poultry by-product meal on the growth, tissue composition and hematological parameters of largemouth bass (*Micropterus salmoides*) fed diets containing different lipids. Aquaculture 260:221–231.

[63] Zhou Q, Zhao J, Li P, Wang H, Wang L (2011). Evaluation of poultry byproduct meal in commercial diets for juvenile cobia (*Rachycentron canadum*). Aquaculture 323:122–127.

[64] Twibell RG, Gannam AL, Hyde NM, Holmes JSA, Poole JB (2012). Effects of fish meal- and fish oil-free diets on growth responses and fatty acid composition of juvenile Coho salmon (*Oncorhynchus kisutch*). Aquaculture 360–361:69–77.

[65] Lee D, Roehm J, Yu TC, Sinnhuber RO. (1967). Effect of ω3 fatty acids on the growth rate of rainbow trout. The Journal of Nutrition 92:93–98. 6067542 10.1093/jn/92.1.93

[66] Castell J, Sinnhuber R, Wales J, Lee D (1972) Essential fatty acids in the diet of rainbow trout (*Salmo gairdnerii*): growth, feed conversion and some gross deficiency symptoms. The Journal of Nutrition 102:77–85. 5007119 10.1093/jn/102.1.77

[67] Sargent JR, Bell JG, McEvoy L, Tocher D, Estevez A (1999). Recent developments in the essential fatty acid nutrition of fish. Aquaculture 177:191–199.

[68] Yu TC, Sinnhuber RO (1979). Effect of dietary ω3 and ω6 fatty acids on growth and feed conversion efficiency of coho salmon (*Oncorhynchus kisutch*). Aquaculture 16(1): 31–38.

[69] Rosenlund G, Torstensen BE, Stubhaug I, Usman N and Sissener NH (2016). Atlantic salmon require long-chain n-3 fatty acids for optimal growth throughout the seawater period. Journal of Nutritional Science 5(e19): 1–13. 10.1017/jns.2016.10 PMC4891698 27293556

[70] Hu H, Kortner TM, Gajardo K, Chikwati E, Tinsley J, Krogdahl Å (2016) Intestinal fluid permeability in Atlantic salmon (*Salmo salar* L.) is affected by dietary protein source. PLoS ONE 11 (12): e0167515 doi: 10.1371/journal.pone.0167515 27907206 10.1371/journal.pone.0167515 PMC5132168

[71] Wong S, Waldrop T, Summerfelt S, Davidson J, Barrows F, Kenney PB, Welch T, et al (2013). Aquacultured rainbow trout (*Oncorhynchus mykiss*) possess a large core intestinal microbiota that is resistant to variation in diet and rearing density. Applied and Environmental Microbiology 79:4974–4984. doi: 10.1128/AEM.00924-13 23770898 10.1128/AEM.00924-13 PMC3754725

[72] Etyemez M, Balcazar JL (2015). Bacterial community structure in the intestinal ecosystem of rainbow trout (*Oncorhynchus mykiss*) as revealed by pyrosequencing-based analysis of 16S rRNA genes. Research in Veterinary Science 100:8–11. doi: 10.1016/j.rvsc.2015.03.026 25843896 10.1016/j.rvsc.2015.03.026

[73] Lyons PP, Turnbull JF, Dawson KA, Crumlish M (2017). Effects of low-level dietary microalgae supplementation on the distal intestinal microbiome of farmed rainbow trout *Oncorhynchus* mykiss (Walbaum). Aquaculture Research 48:2438–2452.

[74] Navarrete P, Magne F, Araneda C, Fuentes P, Barros L, Opazo R, et al (2012). PCR-TTGE analysis of 16S rRNA from rainbow trout (*Oncorhynchus mykiss*) gut microbiota reveals host-specific communities of active bacteria. PloS ONE 7(2):e31335 doi: 10.1371/journal.pone.0031335 22393360 10.1371/journal.pone.0031335 PMC3290605

[75] Bolnick DI, Snowberg LK, Hirsch PE, Lauber CL, Org E, Parks B, et al (2014). Individual diet has sex-dependent effects on vertebrate gut microbiota. Nature Communications 5:4500 doi: 10.1038/ncomms5500 25072318 10.1038/ncomms5500 PMC4279269

[76] Li J, Ni J, Li J, Wang C, Li X, Wu S, et al (2014). Comparative study on gastrointestinal microbiota of eight fish species with different feeding habits. Journal of Applied Microbiology 117:1750–1760. doi: 10.1111/jam.12663 25294734 10.1111/jam.12663

[77] Givens CE, Ransom B, Bano N, Hollibaugh JT (2014). A fish tale: comparison of the gut microbiomes of 12 finfish and 3 shark species. Marine Ecology Progress Series 518:209–223.

[78] Lyons PP, Turnbull JF, Dawson KA, Crumlish M (2016). Phylogenetic and functional characterization of the distal intestinal microbiome of rainbow trout *Oncorhynchus mykiss* from both farm and aquarium settings. Journal of Applied Microbiology 122:347–363. doi: 10.1111/jam.13347 27860093 10.1111/jam.13347

[79] Mansfield GS, Desai AR, Nilson SA, Van Kessel AG, Drew MD, Hill JE (2010). Characterization of rainbow trout (*Oncorhynchus mykiss*) intestinal microbiota and inflammatory marker gene expression in a recirculating aquaculture system. Aquaculture 307:95–104.

[80] Hovda MB, Lunestad BT, Fontanillas R, Rosnes JT (2007). Molecular characterization of the intestinal microbiota of farmed Atlantic salmon (*Salmo salar* L.). Aquaculture 272:581–588.

[81] Roeselers G, Mittge EK, Stephens WZ, Parichy DM, Cavanaugh CM, Guillemin K, et al (2011). Evidence for a core gut microbiota in the zebrafish. ISME J 5:1595–1608. doi: 10.1038/ismej.2011.38 21472014 10.1038/ismej.2011.38 PMC3176511

[82] Kim SK, Bhatnagar I, Kang KH (2012). Development of marine probiotics: prospects and approach. Advances in Food and Nutrition Research 65:353–362. doi: 10.1016/B978-0-12-416003-3.00023-8 22361199 10.1016/B978-0-12-416003-3.00023-8

[83] Askarian F, Kousha A, Salma W, Ringø E (2011). The effect of lactic acid bacteria administration on growth, digestive enzyme activity and gut microbiota in Persian sturgeon (*Acipenser persicus*) and beluga (*Huso huso*) fry. Aquaculture Nutrition 17:488–497.

[84] Balcazar JL., de Blas I, Ruiz-Zarzuela I, Vendrell D, Calvo AC, Marquez I, et al (2007). Changes in intestinal microbiota and humoral immune response following probiotic administration in brown trout (Salmo trutta). Br J Nutr 97:522e7 17313714 10.1017/S0007114507432986

[85] Ringø E, Gatesoupe FJ (1998). Lactic acid bacteria in fish: a review. Aquaculture 160:177e203.

[86] Sun H, Jami E, Harpaz S, Mizrahi I (2013). Involvement of dietary salt in shaping bacterial communities in European sea bass (*Dicentrarchus labrax*). Scientific Reports 3:1558 doi: 10.1038/srep01558 23558231 10.1038/srep01558 PMC3617427

[87] Wong JM, de Souza R, Kendall CW, Emam A, Jenkins DJ (2006). Colonic health: fermentation and short chain fatty acids. Journal of Clinical Gastroenterology 40(3):235–43. 16633129 10.1097/00004836-200603000-00015

[88] Walker AW, Ince J, Duncan SH, Webster LM, Holtrop G, Ze X, et al (2011). Dominant and diet-responsive groups of bacteria within the human colonic microbiota. ISME Journal 5:220–30. doi: 10.1038/ismej.2010.118 20686513 10.1038/ismej.2010.118 PMC3105703

[89] Refstie SL, Sahlstöm S, Bråthen E, Baeverfjord G, Krogedal P (2005). Lactic acid fermentation eliminates indigestible carbohydrates and antinutritional factors in soybean meal for Atlantic salmon (*Salmo salar*). Aquaculture 246: 331–345.

[90] Granada CE, Hasan C, Marder M, Konrad O, Vargas LK, Passaglia LMP, et al (2018). Biogas from slaughterhouse wastewater anaerobic digestion is driven by the archaeal family *Methanobacteriaceae* and bacterial families *Porphyromonadaceae* and *Tissierellaceae*. Renewable Energy 118: 840–846.

[91] Kapatral V, Ivanova N, Anderson I, Reznik G, Bhattacharyya A, Gardner WL, et al (2003). Genome analysis of *F-nucleatum* sub spp *vincentii* and its comparison with then genome of *F-nucleatum* ATCC 25586. Genome Research 13:1180–1189. doi: 10.1101/gr.566003 12799352 10.1101/gr.566003 PMC403646

[92] Mátis G, Neogrády Z, Csikó G, Kulcsár A, Kenéz Á, Huber K (2013). Effects of orally applied butyrate bolus on histone acetylation and cytochrome P450 enzyme activity in the liver of chicken—a randomized controlled trial. Nutrition & metabolism (London) 10:12. 10.1186/1743-7075-10-12 PMC3561214 23336999

[93] Gálfi P, Neogrády S (2002). The pH-dependent inhibitory action of n-butyrate on gastrointestinal epithelial cell division. Food Research International 345:81–586.

[94] Berni Canani R, Di Costanzo M, Leone L, Pedata M, Meli R, Calignano A (2011). Potential beneficial effects of butyrate in intestinal and extra intestinal diseases. World Journal of Gastroenterology 17(12):1519–1528. 21472114 10.3748/wjg.v17.i12.1519 PMC3070119

[95] Vinolo MAR, Ferguson GJ, Kulkarni S, Damoulakis G, Anderson K, Bohlooly YM, et al (2011). SCFAs Induce Mouse Neutrophil Chemotaxis through the GPR43 Receptor. PLoS ONE 6(6):e21205 doi: 10.1371/journal.pone.0021205 21698257 10.1371/journal.pone.0021205 PMC3115979

[96] Toden S, Bird AR, Topping DL, Conlon MA (2007). Dose-dependent reduction of dietary protein-induced colonocyte DNA damage by resistant starch in rats correlates more highly with caecal butyrate than with other short chain fatty acids. Cancer Biology & Therapy 6(2):253–258. 17218781 10.4161/cbt.6.2.3627

[97] Hamer HM, Jonkers D, Venema K, Vanhoutvin S, Troost FJ, Brummer RJ (2008). Review article: the role of butyrate on colonic function. Alimentary Pharmacology & Therapeutics 27:104–119. 17973645 10.1111/j.1365-2036.2007.03562.x

[98] Terova G, Díaz N, Rimoldi S, Ceccotti C, Gliozheni E, Piferrer F (2016). Effects of Sodium Butyrate Treatment on Histone Modifications and the Expression of Genes Related to Epigenetic Regulatory Mechanisms and Immune Response in European Sea Bass (*Dicentrarchus labrax*) Fed a Plant-Based Diet. PLoS ONE 11(7): e0160332 doi: 10.1371/journal.pone.0160332 27471849 10.1371/journal.pone.0160332 PMC4966935

[99] Rimoldi S, Finzi G, Ceccotti C, Girardello R, Grimaldi A, Ascione C, Terova G (2016). Butyrate and taurine exert a mitigating effect on the inflamed distal intestine of European sea bass fed with a high percentage of soybean meal. Fisheries and Aquatic Sciences 19:40.

[100] Savas S, Kubilay A, Basmaz N (2005). Effect of bacterial load in feeds on intestinal microflora of seabream (*Sparus aurata*) larvae and juveniles. Israeli Journal of Aquaculture-Bamidgeh 57:3–9.

