# Supplementary figures and images for: Next generation sequencing for gut microbiome characterization in rainbow trout (Oncorhynchus mykiss) fed animal by-product meals as an alternative to fishmeal protein sources
Source: PLoS One. 2018 Mar 6;13(3):e0193652. doi: 10.1371/journal.pone.0193652 (PMC5839548; doi:10.1371/journal.pone.0193652)

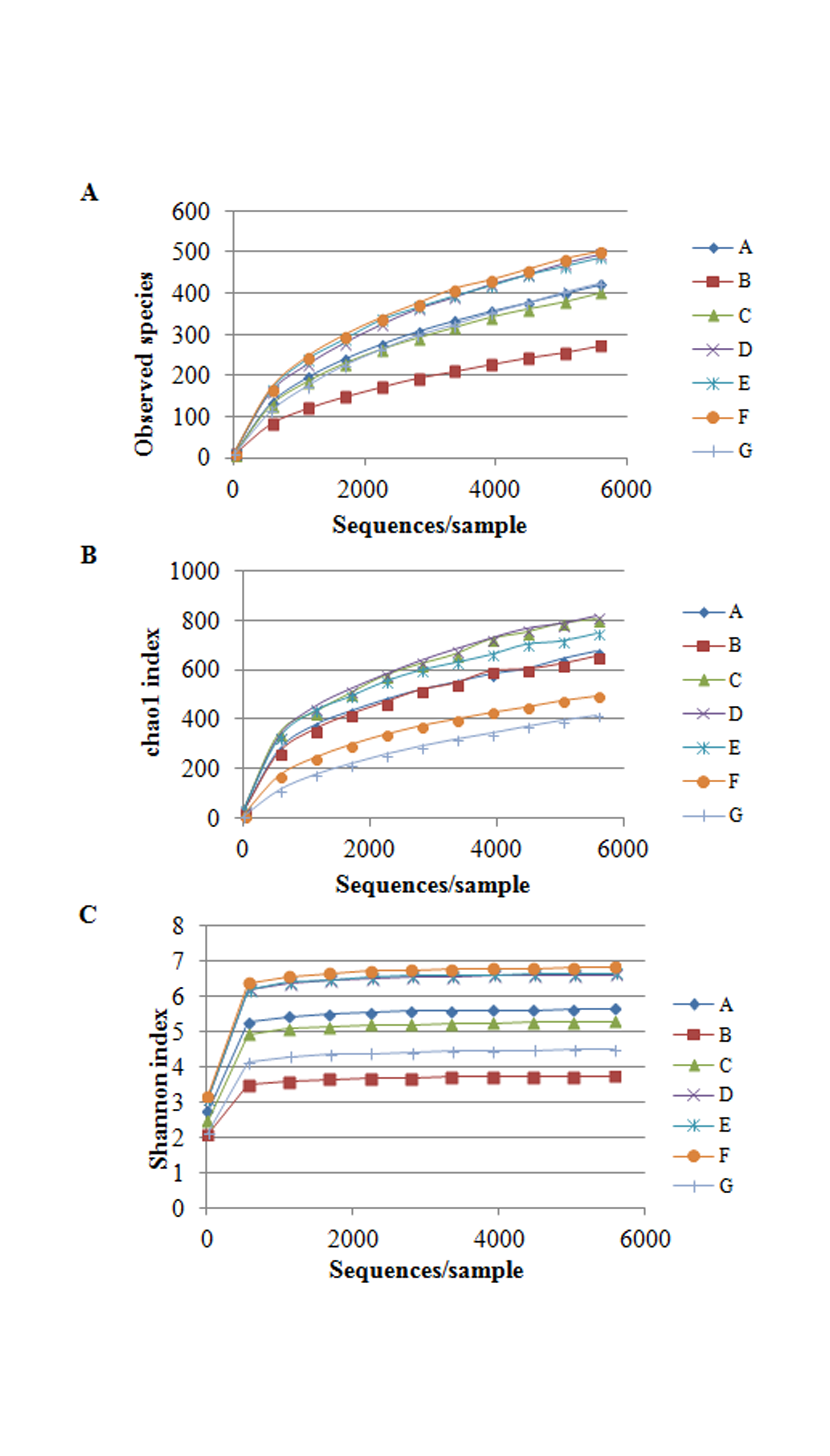

Supplement: S1 Fig — Rarefaction curves of faecal microbial communities from trout fed different diets. (A) Observed species, (B) species richness (Chao1), (C) Shannon’s diversity index. Data points represent the mean values (n = 4). (TIF) [file pone.0193652.s001.tif]

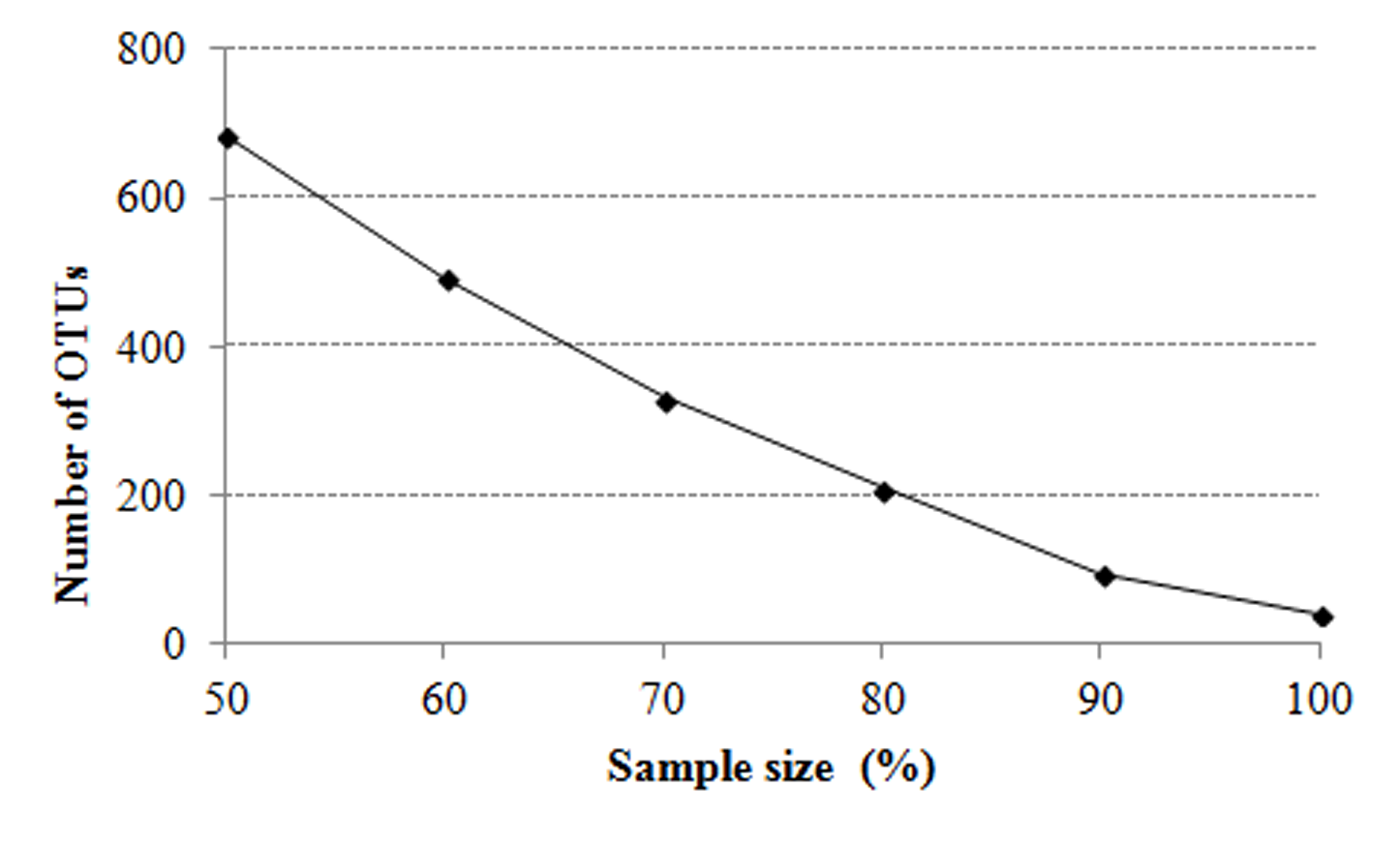

Supplement: S2 Fig — The x-axis represents the percentage of prevalence in all samples (n = 28) regardless of the diet type, the y-axis represents the number of shared OTUs. (TIF) [file pone.0193652.s002.tif]
