# Supplementary material for: Next generation sequencing for gut microbiome characterization in rainbow trout (Oncorhynchus mykiss) fed animal by-product meals as an alternative to fishmeal protein sources
Source: PLoS One. 2018 Mar 6;13(3):e0193652. doi: 10.1371/journal.pone.0193652 (PMC5839548; doi:10.1371/journal.pone.0193652)
Supplement: S1 Table — Significance codes: *P < 0.05, **P < 0.01, ***P < 0.001. (DOCX) [file pone.0193652.s003.docx]

S1 Table

|  | **A:D** | **A:E** | **A:F** | **A:G** | **B:D** | **B:E** | **B:F** | **C:B** | **C:D** | **C:E** | **C:F** | **C:G** | **D:E** | **D:F** | **D:G** | **E:F** | **E:G** | **F:G** |
| --- | --- | --- | --- | --- | --- | --- | --- | --- | --- | --- | --- | --- | --- | --- | --- | --- | --- | --- |
| **Phylum** |  |  |  |  |  |  |  |  |  |  |  |  |  |  |  |  |  |  |
| *Fusobacteria* | ** |  |  |  | *** |  |  |  | * |  |  | * | *** | *** | *** |  |  |  |
| *Bacteroidetes* | * |  |  |  | *** |  |  |  |  |  |  | * | * | *** | *** |  |  |  |
| **Class** |  |  |  |  |  |  |  |  |  |  |  |  |  |  |  |  |  |  |
| *Erysipelotrichia* |  |  | *** |  |  |  | *** |  |  |  | *** |  |  | *** |  | *** |  | *** |
| *Flavobacteriia* | ** |  | ** |  | *** | * | *** |  | ** |  | * |  |  |  | *** |  | ** | *** |
| *Fusobacteriia* | ** |  |  |  | *** |  |  |  | * |  |  | * | *** | *** | *** |  |  |  |
| *Bacteroidia* | * |  |  |  | *** |  |  |  |  |  |  | * | * | *** | *** |  |  |  |
| **Order** |  |  |  |  |  |  |  |  |  |  |  |  |  |  |  |  |  |  |
| *Erysipelotrichales* |  |  | *** |  |  |  | *** |  |  |  | *** |  |  | *** |  | *** |  | *** |
| *Flavobacteriales* | ** |  | ** |  | *** | * | *** |  | ** |  | * |  |  |  | *** |  | ** | *** |
| *Xanthomonadales* | * |  | *** |  | ** |  | *** |  |  |  | *** |  | * |  | ** | *** |  | *** |
| *Fusobacteriales* | ** |  |  |  | *** |  |  |  | * |  |  | * | *** | *** | *** |  |  |  |
| *Bacteroidales* | * |  |  |  | *** |  |  |  |  |  |  | * | * | *** | *** |  |  |  |
| *Enterobacteriales* |  |  | ** |  | * |  | *** |  |  |  | ** |  |  |  |  |  |  | ** |
| *Pasteurellales* | * |  |  |  | ** |  |  |  |  |  |  |  | ** |  | *** |  |  |  |
| **Family** |  |  |  |  |  |  |  |  |  |  |  |  |  |  |  |  |  |  |
| *Enterococcaceae* |  |  | *** |  |  | * | *** |  |  |  | *** |  |  | *** |  | *** |  | *** |
| *Erysipelotrichaceae* |  |  | *** |  |  |  | *** |  |  |  | *** |  |  | *** |  | *** |  | *** |
| *Xanthomonadaceae* | * |  | *** |  | ** |  | *** |  |  |  | *** |  | * |  | ** | *** |  | *** |
| *Fusobacteriaceae* | ** |  |  |  | *** |  |  |  | * |  |  | * | *** | *** | *** |  |  |  |
| *Flavobacteriaceae* | ** |  | * |  | *** |  | ** |  | ** |  | * |  |  |  | *** |  | * | *** |
| *Porphyromonadaceae* | * |  |  |  | ** |  |  |  |  |  |  | * |  | *** | *** |  |  |  |
| *Corynebacteriaceae* | *** |  |  |  | *** |  |  |  | ** |  |  |  | ** | * | *** |  |  |  |
| *[Mogibacteriaceae]* |  |  |  |  |  |  |  | * |  | ** | *** | *** |  | * | * |  |  |  |
| *Bacteroidaceae* | * |  |  |  | ** |  |  |  |  |  |  |  | ** | ** | *** |  |  |  |
| *[Tissierellaceae]* |  |  |  | * | * |  |  | * |  |  | ** | ** |  | * | ** |  |  |  |
| *Enterobacteriaceae* |  |  | ** |  | * |  | *** |  |  |  | ** |  |  |  |  |  |  | ** |
| *Pasteurellaceae* | * |  |  |  | ** |  |  |  |  |  |  |  | ** |  | *** |  |  |  |
| *Streptococcaceae* |  |  |  | * |  |  |  |  |  |  |  | * |  |  | * |  | ** | ** |
| *Pseudomonadaceae* |  |  | * |  |  |  | ** |  |  |  | ** |  |  |  |  |  |  | * |

**S1 Table (continued)**

|  | **A:D** | **A:E** | **A:F** | **A:G** | **B:D** | **B:E** | **B:F** | **C:B** | **C:D** | **C:E** | **C:F** | **C:G** | **D:E** | **D:F** | **D:G** | **E:F** | **E:G** | **F:G** |
| --- | --- | --- | --- | --- | --- | --- | --- | --- | --- | --- | --- | --- | --- | --- | --- | --- | --- | --- |
| **Genus** |  |  |  |  |  |  |  |  |  |  |  |  |  |  |  |  |  |  |
| *Vagococcus* |  |  | *** |  |  |  | *** |  |  |  | *** |  |  | *** |  | *** |  | *** |
| *Lactococcus* |  | *** | *** |  |  | *** | *** |  |  | *** | *** |  | *** | *** |  |  | *** | *** |
| *Erysipelothrix* |  |  | *** |  |  |  | *** |  |  |  | *** |  |  | *** |  | *** |  | *** |
| *Sporanaerobacter* |  | *** |  |  | * | *** |  |  |  | *** |  |  | ** | * | ** | *** | *** |  |
| *Tepidimicrobium* |  | * | * | * | *** |  |  |  |  | * | * | * | *** | *** | *** |  |  |  |
| *Wohlfahrtiimonas* |  |  | *** |  | ** |  | *** |  |  |  | *** |  | * |  | ** | *** |  | *** |
| *Fusobacterium* | ** |  |  |  | *** |  |  |  | * |  |  | * | *** | *** | *** |  |  |  |
| *Granulicatella* |  |  | *** |  |  |  | *** |  |  |  | ** |  |  | *** |  | *** |  | *** |
| *Porphyromonas* | * |  |  |  | ** |  |  |  |  |  |  | * |  | *** | *** |  |  |  |
| *Myroides* | ** |  |  |  | *** |  | * |  | ** |  |  |  |  |  | *** |  | * | ** |
| *Proteus* |  |  | ** |  | ** |  | *** |  | * |  | ** |  | * |  | ** | ** |  | *** |
| *Helcococcus* |  |  |  |  | * |  |  | * |  | * | ** | ** | * | ** | ** |  |  |  |
| *Corynebacterium* | *** |  |  |  | *** |  |  |  | ** |  |  |  | ** | * | *** |  |  |  |
| *Bacteroides* | * |  |  |  | ** |  |  |  |  |  |  |  | ** | ** | *** |  |  |  |
| *pH2* |  |  |  |  |  |  |  | ** | * | ** | *** | *** |  |  |  |  |  |  |
| *Enterococcus* |  |  | ** |  |  |  | *** |  |  |  | ** |  |  | ** |  |  |  | * |
| *Pasteurella* | * |  |  |  | ** |  |  |  |  |  |  |  | ** |  | *** |  |  |  |
| *Peptoniphilus* |  |  |  | * | * |  |  |  |  |  | * | * |  | * | ** |  |  |  |
